# A massive update of non-indigenous species records in Mediterranean marinas

**DOI:** 10.7717/peerj.3954

**Published:** 2017-10-24

**Authors:** Aylin Ulman, Jasmine Ferrario, Anna Occhpinti-Ambrogi, Christos Arvanitidis, Ada Bandi, Marco Bertolino, Cesare Bogi, Giorgos Chatzigeorgiou, Burak Ali Çiçek, Alan Deidun, Alfonso Ramos-Esplá, Cengiz Koçak, Maurizio Lorenti, Gemma Martinez-Laiz, Guenda Merlo, Elisa Princisgh, Giovanni Scribano, Agnese Marchini

**Affiliations:** 1 Department of Earth and Environmental Sciences, University of Pavia, Pavia, Italy; 2 Laboratoire d’Ecogéochimie des Environnements Benthiques, Université Pierre et Marie-Curie, Banyuls-sur-Mer, France; 3 Institute of Marine Biology, Biotechnology and Aquaculture, Hellenic Center of Marine Research, Heraklion, Crete, Greece; 4 Dipartimento di Scienze della Terra dell’Ambiente e della Vita (DISTAV), Università degli Studi di Genova, Genova, Italy; 5 Gruppo Malacologico Livornese, Livrono, Italy; 6 Department of Biological Sciences, Eastern Mediterranean University, Famagusta, North Cyprus, via Mersin 10, Turkey; 7 Department of Geosciences, University of Malta, Msida, Malta; 8 Marine Research Centre (CIMAR), University of Alicante, Alicante, Spain; 9 Department of Hydrobiology, Faculty of Fisheries, Ege University, Izmir, Turkey; 10 Center of Villa Dohrn-Benthic Ecology, Stazione Zoologica Anton Dohrn, Ischia, Italy; 11 Department of Zoology, University of Seville, Seville, Spain

**Keywords:** Alien species, Expansion, Distribution, Macroinvertebrates, New records, Pathways, Recreational boating, Vectors

## Abstract

The Mediterranean Sea is home to over 2/3 of the world’s charter boat traffic and hosts an estimated 1.5 million recreational boats. Studies elsewhere have demonstrated marinas as important hubs for the stepping-stone transfer of non-indigenous species (NIS), but these unique anthropogenic, and typically artificial habitats have largely gone overlooked in the Mediterranean as sources of NIS hot-spots. From April 2015 to November 2016, 34 marinas were sampled across the following Mediterranean countries: Spain, France, Italy, Malta, Greece, Turkey and Cyprus to investigate the NIS presence and richness in the specialized hard substrate material of these marina habitats. All macroinvertebrate taxa were collected and identified. Additionally, fouling samples were collected from approximately 600 boat-hulls from 25 of these marinas to determine if boats host diverse NIS not present in the marina. Here, we present data revealing that Mediterranean marinas indeed act as major hubs for the transfer of marine NIS, and we also provide evidence that recreational boats act as effective vectors of spread. From this wide-ranging geographical study, we report here numerous new NIS records at the basin, subregional, country and locality level. At the basin level, we report three NIS new to the Mediterranean Sea (*Achelia sawayai sensu lato*, *Aorides longimerus*, *Cymodoce* aff. *fuscina*), and the re-appearance of two NIS previously known but currently considered extinct in the Mediterranean (*Bemlos leptocheirus, Saccostrea glomerata*). We also compellingly update the distributions of many NIS in the Mediterranean Sea showing some recent spreading; we provide details for 11 new subregional records for NIS (*Watersipora arcuata*, *Hydroides brachyacantha sensu lato* and *Saccostrea glomerata* now present in the Western Mediterranean; *Symplegma brakenhielmi*, *Stenothoe georgiana*, *Spirobranchus tertaceros sensu lato*, *Dendostrea folium sensu lato* and *Parasmittina egyptiaca* now present in the Central Mediterranean, and *W. arcuata*, *Bemlos leptocheirus* and *Dyspanopeus sayi* in the Eastern Mediterranean). We also report 51 new NIS country records from recreational marinas: 12 for Malta, 10 for Cyprus, nine for Greece, six for Spain and France, five for Turkey and three for Italy, representing 32 species. Finally, we report 20 new NIS records (representing 17 species) found on recreational boat-hulls (mobile habitats), not yet found in the same marina, or in most cases, even the country. For each new NIS record, their native origin and global and Mediterranean distributions are provided, along with details of the new record. Additionally, taxonomic characters used for identification and photos of the specimens are also provided. These new NIS records should now be added to the relevant NIS databases compiled by several entities. Records of uncertain identity are also discussed, to assess the probability of valid non-indigenous status.

## Introduction

The seas are being rapidly being tainted by many harmful stressors such as climate change, overfishing, pollution and non-indigenous species (NIS) (Occhipinti-Ambrogi, 2007). The Mediterranean recreational boating fleet is estimated to contain approximately 1.5 million vessels and hosts over 70% of global charter boating traffic (Cappato, 2011). It is also the world’s most invaded sea, hosting over 700 NIS (Galil et al., 2017), over half of which have Indo-Pacific origins and have probably arrived via the Suez Canal (Galil, Marchini & Occhipinti-Ambrogi, in press). The human-mediated transport of species across boundaries is dramatically altering the natural distribution of marine biota, impacting biodiversity as well as human well-being (Carlton, 1989; Occhipinti-Ambrogi, 2007).

Biological invasions are not only important to understand due to their associated ecological and economic impacts; but they also provide an opportunity to understand other important biogeographic processes such as long-distance dispersal, rapid adaptation and range-expansion processes (Viard, David & Darling, 2016). To properly assess the bioinvasion process and understand the scale of the associated threats, it is first necessary to have the most up-to-date information regarding species distributions, which are used to feed the many databases such as the European Alien Species Information Network (Katsanevakis et al., 2015), the World Register of Introduced Alien Species (Pagad et al., 2017) and AquaNIS-Information system on aquatic NIS and cryptogenic species (Olenin et al., 2014). These databases are highly utilized by scientists and legislators wishing to assess the breadth of the ecological and socio-economic consequences of biological invasions by understanding species’ distributions, measuring trends, and generating ecological models.

Most records of NIS in the Mediterranean Sea originate from occasional or casual findings, while only a few monitoring programs thus far have specifically targeted Mediterranean marine NIS, mainly addressing Marine Protected Areas (MPAs, Mannino et al., 2017), commercial harbors (López-Legentil et al., 2015), or aquaculture sites (Verlaque, 2001). Recreational marinas have not yet been systematically surveyed in the Mediterranean, despite the recent international literature indicating they are important hubs for new species introduction and secondary spreading events (Acosta & Forrest, 2009; Floerl et al., 2009; Clarke-Murray, Pakhamov & Therriault, 2011; Ashton, Davidson & Ruiz, 2014). Furthermore, several recent records of marine NIS in the Mediterranean come from marina habitats (Ros et al., 2013; Marchini, Ferrario & Minchin, 2015; Marić et al., 2016; Ferrario et al., 2017; Steen et al., 2017), suggesting that marinas are part of the stepping-stone invasion process.

The definition of NIS adopted here is: “An organism introduced outside its natural past or present distribution range by direct or indirect human activity (European Environment Agency, 2012).” This definition implies an anthropogenic-assisted transport via various pathways, albeit intentional or unintentional. The route that a new species is transported through to a recipient region is treated as a “pathway.” In the Mediterranean Sea, in addition to shipping and aquaculture (together considered the principal pathways of global NIS introductions), the Suez Canal is frequently referenced as another relevant pathway for the migration of Indo-Pacific species (Galil et al., 2017), and references therein). Each of these “pathways” can have several “vectors” attributed to them, which is the means by which they were transported (Minchin et al., 2009; Olenin et al., 2014). For example, the “shipping” pathway can have the following associated transport vectors: hull-fouling, ballast water and sea chests. There is a high level of uncertainty associated with many of these pathways and vectors since it is rather impossible to prove how a species had been transported, although inferential reasoning on the locality, and proximity to known hubs for NIS introductions such as major ports, aquaculture farms or the Suez Canal make it possible to put forth scientifically sound hypotheses. For this reason, a NIS is often defined as “polyvectic species” *sensu* Carlton & Ruiz (2005, see definitions), because it could have been introduced by a certain combination of pathways or vectors.

This contribution presents new records from the first large-scale survey of Mediterranean marinas for NIS. From April 2015 to November 2016, 34 marinas were sampled for NIS across the Mediterranean spanning from Spain, France, Italy, Malta, Greece, Turkey and Cyprus. Additionally, when permitted, boat-hulls were also inspected for NIS and their captains interviewed about the boats recent travel history since its last hull-cleaning to investigate if recreational boats indeed do seed new NIS propagules to marinas they are visiting, i.e., to verify the role that recreational boating plays as a vector of spread of NIS. Here, we present new NIS records either for the Mediterranean basin, sub-region, country or locality. The new records are presented by taxa, with information on the native origin of the species, their global and Mediterranean distributions, and details of the present record. Here, new records are provided for 32 macroinvertebrate species in Mediterranean marinas and an additional six species found on boat-hulls but not in the marina.

## Materials and Methods

**Figure 1:**
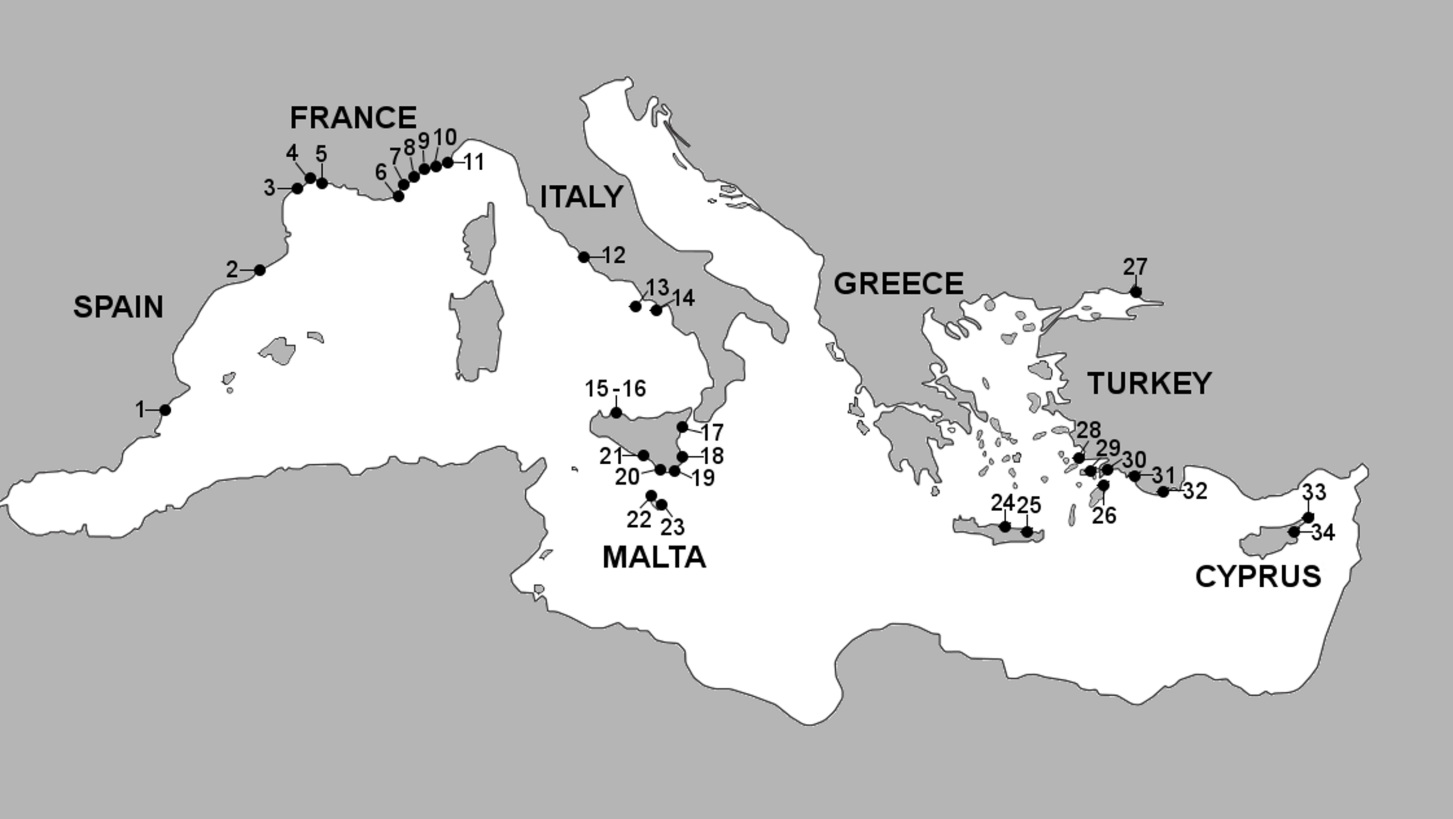
Marinas sampled in this study. Marina numbers correspond to marinas listed in Table 1.

The criteria used for marina selection initially included the sub-region to which they belong, the number of berths (marina size) and popularity as a tourist locality, and in addition, the possibility of obtaining permissions and feasibility. The number of visiting vessels to each marina per annum and staying at least one night was also meant to be used as a proxy for marina selection, however these data were only available for 20 marinas (Table S1). A total of 34 marinas were sampled in seven countries, along with a subset of recreational boat-hulls from 25 of the marinas (Table 1; Fig. 1).

**Table 1:** List of marinas sampled, with corresponding number, geographical coordinates, sampling dates and boat hulls sampled or not (Y, Yes; N, No).

Country	#	Locality name	Marina name	Latitude and longitude	Sampling dates	Boats sampled
**Spain**	1	Alicante	Marina de Alicante	38.339°N; 0.4799°E	14/11/2016	N
	2	Barcelona	One Ocean Port Vell	41.376°N; 2.187°E	22/11/2016	N
**France**	3	Agde	Port Principal du Cap d’Agde	43.281°N; 3.501°E	5–18/06/2015	Y
	4	La Grande-Motte	Port de la Grande-Motte	43.557°N; 4.082°E	02/11/2016	N
	5	Le Grau-du-Roi	Port du Plaisance du Port Camargue	43.515°N; 4.132°E	16–28/05/2015	Y
	6	Saint-Tropez	Port de Saint-Tropez	43.278°N; 6.637°E	1–30/04/2016	Y
	7	Cogolin	Marines de Cogolin	43.065°N; 6.586°E	1–30/04/2016	Y
	8	Sainte-Maxime	Port Privé de Sainte-Maxime	43.307°N; 6.638°E	1–30/04/2016	Y
	9	Cannes	Cannes Le Vieux Port	43.540°N; 7.032°E	19–28/04/2015	Y
	10	Antibes	Port Vauban	43.585°N; 7.127°E	1–12/05/2015	Y
	11	Villefranche-sur-Mer	Port de Villefranche	43.698°N; 7.307°E	22–30/11/2016	N
**Italy**	12	Lido di Ostia	Porto Turistico di Roma	41.737°N; 12.250°E	12–19/07/2015	Y
	13	Ischia Island	Marina di Casamicciola; Marina di Sant’Angelo; Porto d’Ischia	40.748°N; 13.906°E 40.695°N; 13.893°E 40.743°N; 13.939°E	1–11/08/2015	Y
	14	Sorrento	Porto Turistico Marina Piccola Sorrento	40.629°N; 14.375°E	22–29/07/2015	Y
	15	Palermo	Marina Villa Igiea	38.142°N; 13.370°E	26–29/07/2016	Y
	16	Palermo	Porto La Cala	38.120°N; 13.368°E	2–3/08/2016	N
	17	Riposto	Porto dell’Etna	37.732°N; 15.208°E	17–28/09/2016	Y
	18	Siracusa	Porto Grande (Marina Yachting)	37.063°N; 15.284°E	15-16/08/2016	N
	19	Marzamemi	Marina di Marzamemi	36.733°N; 15.119°E	08/10/2016	N
	20	Marina di Ragusa	Porto Turistico Marina di Ragusa	36.781°N; 14.546°E	1–7/09/2016	Y
	21	Licata	Marina di Cala del Sole	37.097°N; 13.943°E	5–10/08/2016	Y
**Malta**	22	Msida	Msida Yacht Marina	35.896°N; 14.493°E	1–8/07/2016	Y
	23	Valletta	Grand Harbor Marina	35.890°N; 14.523°E	11–18/07/2016	Y
**Greece**	24	Heraklion	Old Venetian Harbor	35.343°N; 25.136°E	1–15/11/2015	Y
	25	Agios Nikolaos	Agios Nikolaos Marina	35.187°N; 25.136°E	18–25/11/2015	Y
	26	Rhodes	Mandraki Port	36.449°N; 28.226°E	2–11/06/2016	Y
**Turkey**	27	Istanbul	Setur Kalamış Marinas	40.976°N; 29.039°E	28/08/2015	Y
	28	Bodrum	Milta Bodrum Marina	37.034°N; 27.425°E	9–11/09/2015	Y
	29	Datça	Datça Marina	26.722°N; 27.689°E	10/10/2015; 13/05/2016	N
	30	Marmaris	Setur Marmaris Netsel Marina	36.852°N; 28.276°E	14–18/09/2015	Y
	31	Fethiye	Eçe Marina	36.623°N; 29.101°E	19–24/09/2015	Y
	32	Finike	Setur Finike Marina	36.294°N; 30.149°E	18–27/05/2016	Y
**Cyprus**	33	Karpaz	Karpaz Gate Marina	35.558°N; 34.232°E	21–27/06/2016	Y
	34	Famagusta	Famagusta Port	35.123°N; 33.952°E	13–19/06/2016	Y

When reporting new sub-regional records for the Mediterranean, the sub-regions included the following sampled countries: Western Mediterranean (Spain and France); Central Mediterranean (Italy and Malta); and Eastern Mediterranean (Greece, Turkey, including the Turkish part of the island of Cyprus thereafter, only “Cyprus” for simplicity).

### Marina substrate sampling

We adopted a rapid assessment protocol (Pedersen et al., 2003; Cohen, 2005; Ashton et al., 2006) targeting all fouling macroinvertebrate taxa. These “rapid assessments” typically target a predefined list of species, involve an onsite team of experts, and generally last an hour. Since our strategy also targeted any unknown invertebrate species for collection, and generally had one or two collectors at most, we increased the typical sampling time to approximately eight hours per marina to allow for the careful collection and sorting of all unknown taxa to ensure no new species could additionally be collected. Samples were taken from each of the central, outer and inner portions of the marinas to guarantee representative samples. For the larger marinas (i.e., Port Camargue, Cap d’Agde), the management authorities provided the use of an inflatable boat with a captain to access hard-to-reach areas, and to ensure the substrate sampling could be completed in one day. Photos were taken of the non-indigenous biota using either a SONY RXIII (with a Nauticam housing) camera, or the Olympus Tough TG-3 or TG-4.

A specialized hand-held rigid net with one sharpened edge was built for the marina substrate sampling (1 mm mesh size, surface 25 × 20 cm), which extended to a depth of 1.5 m from the pier to scrape the submerged areas of the pontoons and marina walls over an area of approximately 0.23 m^2^. Ladders, tires and buoys were also scraped using a 6.35 cm (diameter) paint scraper or manually. Next, the biota were placed in a plastic tray and immediately sorted to major taxonomic groups into smaller bottles containing a 75–90% ethanol solution (such as crustaceans, molluscs, polychaetes, etc.) for further laboratory analysis. The smaller-sized taxa were filtered and collected using a sieve with a 1 mm mesh size. The only exception were the ascidians, which were immediately preserved in seawater, then later placed in a freezer for 30–90 min (with care taken not to freeze the sample), then transferred to a 4% formalin/seawater solution for 48 h for fixing, and lastly preserved in a 90% ethanol solution; this procedure is necessary for maintaining some rigidity in the specimens structure, necessary for dissection.

### Boat-hull sampling

A preliminary screening was first completed with boat captains/owners before selection to ensure that their boat had travelled outside the marina in the past 12 months for a minimum of one night, so that the vessel posed some risk of spreading NIS. Next, and only with permission from the boat owners/captains, fouling samples were collected from the boat-hulls and a short survey was completed with the boat owners/captains on the vessel’s characteristics, hull-cleaning and painting details, and recent 12 months of travel history. The fouling samples were collected from the boat-hulls using one of three approaches (which were dependent on authorizations and feasibility): The first approach involved inspecting the boat-hulls immediately as they were hoisted from the water at the *carénage* (haul-out station) for their maintenance routines (cleaning/painting/repairs). This approach was mainly used in France: Cannes, Antibes, Marines de Cogolin, Saint Maxime, Saint Tropez and Cap d’Agde as the sampling season provided the optimal opportunity to use this approach as these routine maintenance procedures normally occur before the onset of the tourist season, and was used sporadically in other marinas only when the opportunity presented itself. The boat-hull including niche areas such as the propeller, propeller shaft, water vents, rudders and ladders were closely inspected and fouling samples were collected using a paint scraper and aquarium fishing net wherever fouling biota were found, and quickly transferred to a bottle containing 90% ethanol. Photographs were also taken from each boat-hull to crosscheck the results and reduce likelihood of mistakes. The remaining boat-hulls were sampled via snorkeling or, on a few occasions, by scuba diving but using the same methods as described above. Care was taken to ensure the sampling strategy did not release NIS propagules into the marina’s waters by scraping the samples collected in-water directly into small, finely meshed aquarium nets. All fouling samples were collected by the first author, with the exception of the boats in Porto Turistico di Roma which were collected by the ports professional scuba diver, with careful instructions from the first author on what to collect after reviewing underwater detailed photos of the hulls.

### Taxonomic identification

This study focused on fouling invertebrates; plants and algae were not examined. All macronivertebrate taxa were collected for identification, and samples requiring expert identification were sent to appropriate experts.

The preserved specimens were observed under a dissecting microscope and, where needed, taxonomic slides were prepared and analyzed under an optical microscope. Photographs of magnified specimens or morphological parts were taken directly from the microscopes using the Olympus TG-4 camera (i.e., for serpulids and crustaceans), or with the Tescan Field Emission Scanning Electron Microscope series Mira 3XMU for SEM pictures, with increasing magnification, at 6–19 mm working distance, using an accelerating voltage of 10 kV, with graphite metallization and detection by secondary electrons (i.e., for bryozoans). Bryozoan specimens used for SEM pictures were cleaned beforehand using a combination of bleach and hydrogen peroxide to remove organic residues. Ascidians were stained with Masson’s haemalum for dissection.

Some of our records refer to species completely new to the Mediterranean Sea, whose taxonomic identity has been verified morphologically, but still requiring further genetic confirmation, since they pertain to taxonomically challenging taxa which have often revealed complexes of cryptic species. Moreover, a couple of our findings include species not yet properly described scientifically; thus it is not possible to assign a certain identification until formal descriptions are completed. These records are discussed in detail to verify the likeliness of representing introduced populations of NIS. To assign a NIS status for such species, the Chapman & Carlton (1991) criteria were followed taking into account factors such as: “appearance in local regions where not found previously;” “association with human mechanisms of dispersal;” “prevalence or restrictions to artificial environments;” “insufficient active or passive dispersal capability” and “exotic evolutionary origin.” Records of species found only on boat-hulls but not in marinas should only be considered as new NIS country records if certain that the boat did not leave that country’s waters, since boats represent mobile habitats and are hence affected by an “uncertain occurrence” (see Marchini, Galil & Occhipinti, 2015).

Non-indigenous species status is dependent on their establishment success in a new locality, and can be defined as either: not established (a single specimen reported in one or two localities, rare, uncommon), or established (evidence of a reproducing population in one or more localities, common or abundant). Additionally, a couple of cases are presented here for “pseudoindigenous species” (see definitions).

## Results

**Figure 2:**
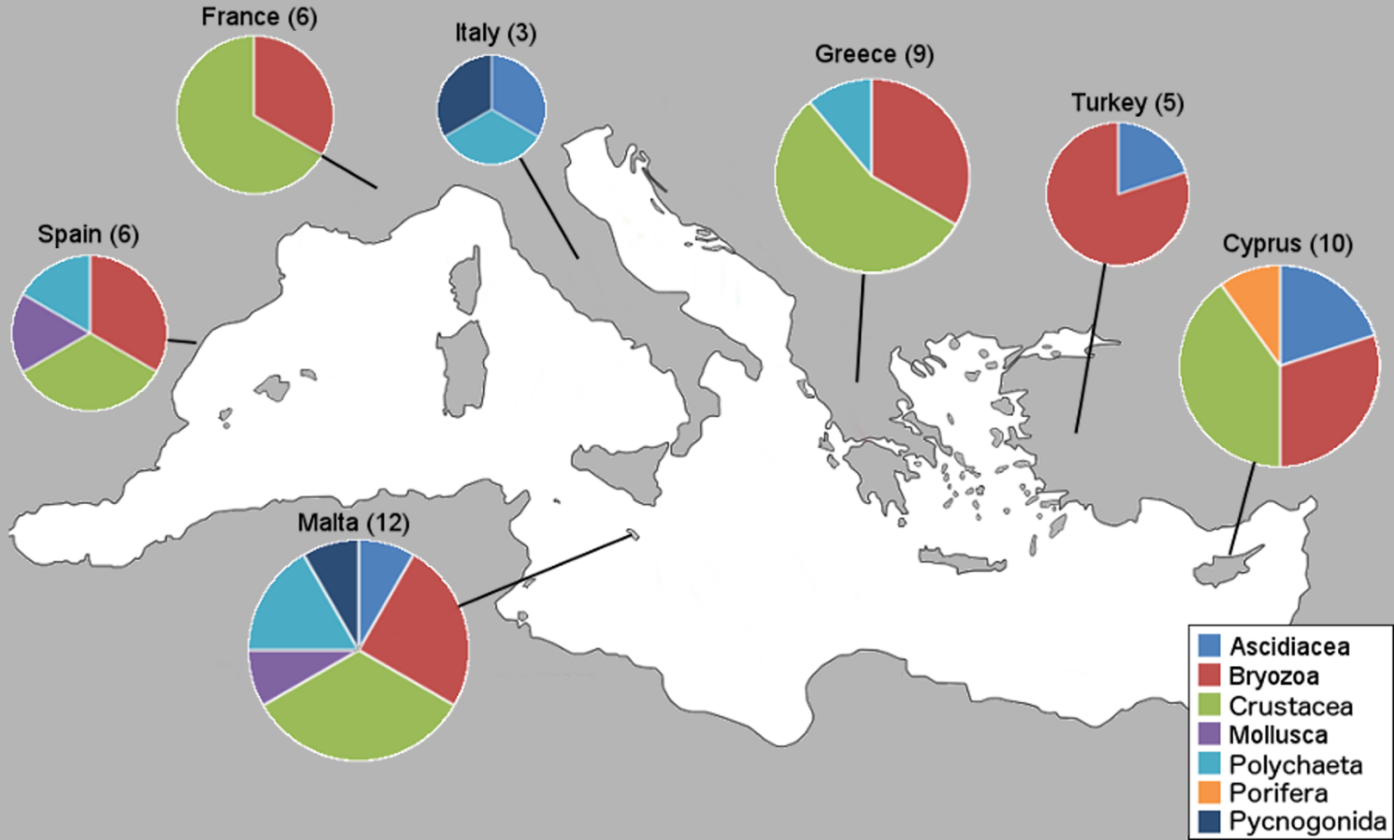
Number of new NIS records per country by taxonomic group. Sampled countries are shown with their number of new NIS records found in this study (in brackets), and pie graphs show the new records represented proportionally by taxa.

Within the framework of this study, a total of 76 NIS were collectively identified from 34 marinas from the seven countries; however, only new country records and interesting new locality records are presented here. First, we present the number of new NIS found in this study per country and by taxa (Fig. 2).

This study revealed three species new to the Mediterranean basin (*Achelia sawayai sensu lato*, *Aoroides longimerus*, and *Cymodoce* aff. *fuscina*), 11 new subregional records (*W. arcuata*, *H. brachyacantha sensu lato* and *Saccostrea glomerata* now present in the Western Mediterranean; *Symplegma brakenhielmi*, *Stenothoe georgiana*, *Spirobranchus tertaceros sensu lato*, *Dendostrea folium sensu lato* and *Parasmittina egyptiaca* now present in the Central Mediterranean, and *W. arcuata*, *Bemlos leptocheirus* and *Dyspanopeus sayi* in the Eastern Mediterranean), for an overall number of 51 new country records and a few new locality records exhibiting distribution expansions. These new Mediterranean basin and country records are presented (Table 2) with the corresponding marina numbers in which they were found from Table 1. Additionally, NIS found on boat-hulls but not in the respective marina, locality or country, are presented as a warning signal for future monitoring (Table 3). The numbers of new NIS found per marina are shown (Table 4), and also the new NIS records are presented by country, specifically 12 for Malta, 10 for Cyprus, nine for Greece, six for Spain and France, five for Turkey and three for Italy (Table 5). Subsequently, all new NIS records are discussed by species (first ordered by class and family, and then alphabetically by species, see “New NIS records: notes on individual species” below). The key taxonomic characters used to identify these species are accompanied as “Supplementary Data,” along with identification photos taken of our specimens. Comprehensive reviews of global and Mediterranean distributions for all NIS listed in Tables 2 and 3 are presented below, along with details on the new record type and if they were found in the marina, on a boat-hull or both.

**Table 2:** New NIS record table by species, with numbers in locality corresponding to marina details from Table 1.

Family	Species	Country and Marina #	Record type
Ascidea	*Clavelina oblonga*	Cyprus (#34)	*
	*Clavelina oblonga*	Turkey (#29)	*
	*Phallusia nigra*	Cyprus (#33, #34)	*
	*Styela plicata*	Malta (#22, #23)	*
	*Symplegma brakenhielmi*	Italy (#15)	*, CM
Bryozoa	*Amathia verticillata*	Malta (#22, #23)	*
	*Amathia verticillata*	Cyprus (#34)	*
	*Amathia verticillata*	Turkey (#28, #30)	*
	*Celleporaria brunnea*	Spain (#1)	*
	*Celleporaria brunnea*	France (#4, #5, #6, #8)	*
	*Celleporaria brunnea*	Malta (#22, #23)	*
	*Celleporaria brunnea*	Greece (#24)	*
	*Celleporaria vermiformis*	Greece (#24, #25, #26)	*
	*Celleporaria vermiformis*	Cyprus (#33, #34)	*
	*Hippopodina* sp. A	Turkey (#32)	*
	*Parasmittina egyptiaca*	Turkey (#32)	*
	*Parasmittina egyptiaca*	Cyprus (#33)	*
	*Tricellaria inopinata*	France (#3, #5)	*
	*Tricellaria inopinata*	Greece (#24)	*
	*Watersipora arcuata*	Spain (#1, #2)	*
	*Watersipora arcuata*	Malta (#22)	*, CM
	*Watersipora arcuata*	Turkey (#28, #32)	*, EM
Crustacea	*Ampithoe bizseli*	Cyprus (#33, #34)	*
	*Aorides longimerus*	France (#5)	**
	*Bemlos leptocheirus*	Greece (#24, #25)	*, EM
	*Charybdis* (*Gonioinfradens*) *paucidentatus*	Cyprus (#34)	*
	*Cymodoce* cf. *fuscina*	Greece (#24)	**
	*Dyspanopeus sayi*	Greece (#24)	*, EM
	*Erichthonius* cf. *pugnax*	France (#5)	*
	*Ianiropsis serricaudis*	France (#3, #5)	*
	*Mesanthura* cf. *romulea*	Spain (#1)	*
	*Mesanthura* cf. *romulea*	Malta (#22)	*
	*Mesanthura* cf. *romulea*	Greece (#26)	*
	*Mesanthura* cf. *romulea*	Cyprus (#33, #34)	*
	*Paracerceis sculpta*	Malta (#22, #23)	*
	*Paracerceis sculpta*	Cyprus (#34)	*
	*Paranthura japonica*	Spain (#1, #2)	*
	*Paranthura japonica*	Malta (#23)	*
	*Sphaeroma walkeri*	Greece (#24)	*
	*Stenothoe georgiana*	France (#5)	*
	*Stenothoe georgiana*	Malta (#23)	*, CM
Mollusca	*Arcuatula senhousia*	Spain (#2)	*
	*Dendostrea folium s.l*.	Malta (#22, #23)	*, CM
Polychaeta	*Hydroides brachyacantha s.l*.	Spain (#2)	*, WM
	*Hydroides brachyacantha s.l*.	Greece (#24)	*
	*Hydroides dirampha*	Malta (#22, #23)	*
	*Hydroides elegans*	Malta (#22)	*
	*Spirobranchus tetraceros s.l*.	Italy (#18)	*, CM
Porifera	*Paraleucilla magna*	Cyprus (#34)	*
	*Achelia sawayai s.l*.	Malta (#23)	**
Pycnogonida	*Achelia sawayai s.l*.	Italy (#17, #18)	**

**Note:**

Record type: *New country record, **New Mediterranean record; Letters indicate a new subregional record (WM, Western Med.; CM, Central Med.; EM, Eastern Med.).

**Table 3:** NIS found on boat hulls but not found in the marina or country.

Family	Species	Country and Marina #	Record type
Ascidiacea	*Clavelina oblonga*	Cyprus (#33)	Λ
Bryozoa	*Amathia verticillata*	Turkey (#31)	Λ
	*Celleporaria brunnea*	France (#3, #7, #9, #10)	Λ
	*Celleporaria brunnea*	Greece (#25, #26)	*
	*Tricellaria inopinata*	Turkey (#27)	*
	*Parasmittina egyptiaca*	Italy (#21)	*, CM
	*Parasmittina egyptiaca*	Greece (#25)	*
	*Watersipora arcuata*	France (#7)	*
Crustacea	*Amphibalanus improvisus*	France (#5)	*
	*Balanus trigonus*	Cyprus (#33)	*
	*Cymodoce* cf. *fuscina*	Greece (#25)	Λ
	*Ericthonius* cf. *pugnax*	France (#3)	Λ
	*Paracerceis sculpta*	Turkey (#31)	*
	*Paradella dianae*	Italy (#20)	Λ
	*Paradella dianae*	Greece (#24)	*
	*Sphaeroma walkeri*	Greece (#25)	Λ
	*Stenothoe georgiana*	France (#3, #10)	Λ
Mollusca	*Dendostrea folium s.l*.	Italy (#17)	*
	*Saccostrea glomerata*	France (#10)	*, WM
Polychaeta	*Hydroides homoceros*	Cyprus (#33)	*

**Note:**

Λ = Not previously known from the locality, * = Not previously known from the country. Letters indicate a new subregional record (WM, Western Med.; CM, Central Med.; EM, Eastern Med.).

**Table 4:** Number of NIS per sampled marina, using marina numbers given in Table 1.

#. Marina locality and country	# NIS	#. Marina locality and country	# NIS
1. Alicante, Spain	10	18. Siracusa, Italy	16
2. Barcelona, Spain	11	19. Marzememi, Italy	11
3. Cap d’Agde, France	8	20. Ragusa, Italy	14
4. La Grand-Motte, France	7	21. Licata, Italy	11
5. Port Camargue, France	17	22. Msida, Malta	14
6. Saint-Tropez, France	4	23. Grand Harbor, Malta	13
7. Cogolin, France	6	24. Heraklion, Greece	27
8. Saint-Maxime, France	3	25. Agios Nikolaos, Greece	12
9. Cannes, France	5	26. Rhodes, Greece	16
10. Antibes, France	5	27. Istanbul, Turkey	4
11. Villefranche-sur-Mer, France	2	28. Bodrum, Turkey	12
12. Rome, Italy	9	29. Datça, Turkey	9
13. Ischia, Italy	5	30. Marmaris, Turkey	6
14. Sorrento, Italy	8	31. Fethiye, Turkey	10
15. Villa Igiea, Italy	20	32. Finike, Turkey	14
16. La Cala, Italy	16	33. Karpaz, Cyprus	17
17. Riposto, Italy	13	34. Famagusta, Cyprus	18

**Table 5:** New NIS records by country, with countries ordered from west to east.

Country	Species	Country	Species
Spain	*Celleporaria brunnea*	Greece	*Celleporaria brunnea*
Spain	*Watersipora arcuata*	Greece	*Celleporaria vermiformis*
Spain	*Mesanthura* cf. *romulea*	Greece	*Tricellaria inopinata*
Spain	*Paranthura japonica*	Greece	*Bemlos leptocheirus*
Spain	*Arcuatula senhousia*	Greece	*Cymodoce* cf*. fuscina*
Spain	*Hydroides brachyacantha s.l*.	Greece	*Dyspanopeus sayi*
France	*Celleporaria brunnea*	Greece	*Mesanthura* cf. *romulea*
France	*Tricellaria inopinata*	Greece	*Sphaeroma walkeri*
France	*Aorides longimerus*	Greece	*Hydroides brachyacantha s.l*.
France	*Erichthonius* cf. *pugnax*	Turkey	*Clavelina oblonga*
France	*Ianiropsis serricaudis*	Turkey	*Amathia verticillata*
France	*Stenothoe georgiana*	Turkey	*Hippopodina* sp. A
Italy	*Symplegma brakenhielmi*	Turkey	*Parasmittina egyptiaca*
Italy	*Spirobranchus tetraceros s.l*.	Turkey	*Watersipora arcuata*
Italy	*Achelia sawayai s.l*.	Cyprus	*Clavelina oblonga*
Malta	*Styela plicata*	Cyprus	*Phallusia nigra*
Malta	*Amathia verticillata*	Cyprus	*Amathia verticillata*
Malta	*Celleporaria brunnea*	Cyprus	*Celloporaria vermiformis*
Malta	*Watersipora arcuata*	Cyprus	*Parasmittina egyptiaca*
Malta	*Mesanthura* cf. *romulea*	Cyprus	*Mesanthura* cf. *romulea*
Malta	*Paracerceis sculpta*	Cyprus	*Ampithoe bizseli*
Malta	*Paranthura japonica*	Cyprus	*Charybdis (Gonioinfradens) paucidentatus*
Malta	*Stenothoe georgiana*	Cyprus	*Paracerceis sculpta*
Malta	*Dendostrea folium s.l*.	Cyprus	*Paraleucilla magna*
Malta	*Hydroides dirampha*		
Malta	*Hydroides elegans*		
Malta	*Achelia sawayai s.l*.		

## New Nis Records: Notes on Individual Species

Please note that the numbers used in describing the locality of the new records correspond to the marinas listed in Table 1.

Class: Ascidiacea

Some ascidians whose likely origin is the Northeast Atlantic (i.e., *Clavelina lepadiformis, Ciona intestinalis, Ascidella aspersa* and *Botryllus schlosseri*) have been excluded from this study which focuses exclusively on NIS. Genetic studies have shown that these species include different clades in the Mediterranean, some which can be considered non-native, and in some cases native (Turon et al., 2003; Perez-Portela et al., 2013; Bouchemousse, Bishop & Viard, 2016; Nydam, Giesbrecht & Stephenson, 2017). These cryptogenic species (Carlton, 1996), their origins and status require additional genetic analyses, which exceeds the breadth of the present study, which is based on morphological characters.

Family: Ascidiidae
***Phallusia nigra*** Savigny, 1816

**Potential native origin:** Uncertain, could be from the Red Sea, Indo-Pacific, or Western Atlantic Ocean.

**Distribution:** First recorded and described from the Red Sea (Savigny, 1816), then in the Gulf of Guinea and Angola (Millar, 1965), the Arabian Gulf (Monniot & Monniot, 1997), the Pacific Ocean (Lambert, 2003), Indian Ocean (Abdul Jaffar, Sivakumar & Tamilselvi, 2009), and the Western Atlantic and Caribbean (Van Name, 1945; Bonnet & Rocha, 2011; Vandepas et al., 2015).

In the Mediterranean, it has only been reported in the Eastern Mediterranean from Israel, Lebanon and the Turkish Levantine coast (Çinar et al., 2006; Shenkar, 2008; Izquierdo-Muñoz, Díaz-Valdés & Ramos-Esplá, 2009), and most recently from Greece, specifically from Chalkidiki and Rhodes (Kondilatos, Corsini-Foka & Pancucci-Papadopoulou, 2010; Thessalou-Legaki et al., 2012).

**New records:** This finding represents the first country record for Cyprus (#33 and #34: Fig. S1A).

**Boat-hull records:** Found on one boat-hull moored in Cyprus (#34).

**Notes:** Although its native origin is uncertain, it is considered a NIS in the Mediterranean (Çinar et al., 2006; Shenkar, 2008). Vandepas et al. (2015) highlighted some uncertainty regarding some *Phallusia nigra* Mediterranean records due to resemblances to the also dark, native congeneric tunicate *Phallusia fumigata* (Gruber, 1864), and confirmed the presence of the introduced *Phallusia nigra* in the Eastern Mediterranean basin. For this reason, the morphology of the *Phallusia* specimens collected from Cyprus were carefully compared to specimens of the native *Phallusia fumigata* (found in our own samples from Port Vell, Barcelona).

Family: Clavelinidae
***Clavelina oblonga*** Herdman, 1880

**Native origin:** Western Atlantic US coast and Caribbean Sea.

**Distribution:** Its initial record is from Bermuda (Van Name, 1945). It is hypothesized to be an introduced species to Brazil, first sighted there in 1925 (Rocha, Kremer & Fehlauer-Ale, 2012). In the Eastern Atlantic, it has been reported as NIS in Cape Verde (Hartmeyer, 1912), Senegal (Pérès, 1951), and the Azores (Monniot & Monniot, 1994). It was described in the Mediterranean half a century after its initial record as *Clavelina phlegraea* from southern Italy and Corsica (Salfi, 1929). It was also found in natural habitats on the Iberian Coast, about 100 km west of Gibraltar (Ordóñez et al., 2016).

**New records:** This finding represents a first country record for Turkey (Marina #29) and Cyprus (#34: Fig. S1B), and two new locality records for mainland France (#5, #7).

**Boat-hull records:** Found on boat-hulls moored in Cyprus (#33 and #34).

**Notes:** The species identified earlier as *Clavelina phlegraea* (Salfi, 1929) in the Mediterranean was thought to be a native species, but recent genetic analysis confirmed it as the introduced *Clavelina oblonga* (Ordóñez et al., 2016). In France, it had only previously been reported in Corsica, so these new records from the French mainland indicate its possible expansion along the coast.

Family: Didemnidae
***Diplosoma listerianum*** (Milne-Edwards 1841)

**Native origin:** Northern Sea.

**Distribution:** This species was first described from England but is well known from marinas and harbors worldwide including the Pacific Northwest, Panama, Chile, Japan, Tahiti, Guam, South Africa and Australia (Rocha & Kremer, 2005; Pérez-Portela et al., 2013). In the Mediterranean, its first record was from Italy in 1975 (Lafargue, 1975), and is now widespread throughout European and Mediterranean waters (Millar, 1969; Ramos-Esplá, 1988; Koukouras et al., 1995; Çinar, 2014).

**New records:** This study presents a new locality record for the Turkish Levantine Sea/Mediterranean coast (#31, #32). During this study, it was also found in France (#5), Malta (#23: Fig. S1C), Turkey (#28) and Greece (#24 and #26).

Family: Pyuridae
***Microcosmus exasperatus*** Heller 1878

**Potential native origin:** Unknown.

This species has a broad global distribution from all continental waters, including remote localities such as Hawaii and the Mariana Islands, but does not occur in Antarctica (Nagar & Shenkar, 2016).

In the Mediterranean, it was first reported from south-eastern Tunisia in 1998 (Meliane, 2002; Ramos-Esplá, Izquierdo-Muñoz & Çinar, 2013), then from Lebanon (Bitar, Ocana & Ramos-Esplá, 2007), Israel (Shenkar, 2008), around the Lebanese coast in 2009 (Ramos-Esplá, Izquierdo-Muñoz & Çinar, 2013), the Aegean Sea of Turkey (Ramos-Esplá, Izquierdo-Muñoz & Çinar, 2013), and North-Western Cyprus (Gewing et al., 2016).

**New records:** This study presents a new locality record for Turkey (#29) as the southernmost record for Turkey, and a new locality for Cyprus (#33), illustrating its ongoing expansion.

**Notes:** *Microcosmus exasperatus* and *Microcosmus squamiger* are both present in the Mediterranean, however, they do not overlap in distributions: *Microcosmus squamiger* is present in the Western and Central Mediterranean whereas *Microcosmus exasperatus* is only present in the Eastern Mediterranean (Ramos-Esplá, Izquierdo-Muñoz & Çinar, 2013). Thus, it has been hypothesized these two species invaded via different entrances to the basin: *Microcosmus squamiger* via the Strait of Gibraltar and *Microcosmus exasperatus* via the Suez Canal (Turón, Nishikawa & Rius, 2007; Ramos-Esplá, Izquierdo-Muñoz & Çinar, 2013). Noteworthy is that *Microcosmus exasperatus* was not found in late 2014 in Karpaz Marina, Cyprus (#33) by Gewing et al. (2016) when specifically looking for this species; however, we found it present there in 2016.

***Microcosmus squamiger*** Michaelsen, 1927

**Potential native origin:** Australia.

**Distribution:** Globally, this species is found in the waters of California, South Africa, Hawaii, and the Western Indian Ocean (Mastrototaro & Dappiano, 2008).

In the Mediterranean, it was first reported from Tunisia in 1967 (Monniot, 1981), and is now found throughout the Western Mediterranean (Monniot, 1981; Ramos-Esplá, 1988; Mastrototaro & Dappiano, 2008; Turón, Nishikawa & Rius, 2007) and from the Central Mediterranean: Taranto, Italy and Grand Harbor, Malta (Izquierdo-Muñoz, Díaz-Valdés & Ramos-Esplá, 2009).

**New records:** This finding represents a new locality for Italy around Sicily (#15, #17, #19, #20: Fig. S1D). From this study, it was also found in Spain (#2).

Family: Styelidae
***Styela plicata*** (Lesueur, 1823)

**Potential native origin:** Unknown, cosmopolitan species.

**Distribution:** This species has been reported worldwide (Harant & Vernières, 1933; Van Name, 1945; Pérès, 1951; Tokioka, 1963; Ramos-Esplá, 1988). It is considered a NIS in California (Lambert & Lambert, 2003), Gulf of Mexico (Lambert, 2005), Brazil (Rocha & Kremer, 2005) and the Mediterranean Sea (Maltagliati et al., 2016).

**New records:** This finding represents a new country record for Malta (#22 and #23). From this study, *Styela plicata* is extremely widespread and was found in all sampled marinas aside from #6, #9, #11, #29 and #33.

**Boat-hull records:** Found on boat-hulls moored in the following marinas: France (#3, #5, #7, #10), Italy (#12, #14: Fig. S1E, #15, #21), Malta (#22), Greece (#24, #26), and Turkey (#31, #32).

**Notes:** This is a well-known cosmopolitan hull-fouling species found from many localities across the Atlantic Ocean from Philadelphia (Van Name, 1945) to Senegal (Pérès, 1951). Recent genetic analysis suggests that its wide geographic distribution is attributed to many introductions stemming from human-mediated hull fouling, triggering multiple introduction events (Barros, Rocha & Pie, 2009). Additionally, most records are from artificial substrates or harbours, also supporting the hypothesis of an ongoing invasion (Barros, Rocha & Pie, 2009).

***Symplegma brakenhielmi*** (Michaelson, 1904)

**Potential native origin:** Unknown

**Distribution:** It has been found in Australian waters (Kott, 2004), the Pacific Panamanian coast (Carman et al., 2011), and from the Atlantic in French Guianese waters (Monniot, 2016). In the Mediterranean, it was reported from Israel in the 1950s (as *Symplegma viride* Herdman, 1886), then from Lebanon (Bitar & Kouli-Bitar, 2001; Bitar, Ocana & Ramos-Esplá, 2007), Israel (Shenkar, 2008) and Turkey (Çinar et al., 2006).

**New records:** This study presents a new country record for Italy (#15), and a new Central Mediterranean subregional record. During this study, it was also found in Turkey (#31 and #32: Fig. S1F).

**Boat-hull records:** Found on one boat-hull moored in Turkey (#31).

**Notes:** It is likely that Pérès (1958) is referring to this species under the name *Symplegma viride*. Antoniadou, Gerovasileiou & Bailly (2016), in their recent update of ascidians found in Greek waters, warned of a high-likelihood of a Greek invasion due to its proximity to the Turkish Levantine coast. This study confirms its spread to the Central Mediterranean. Soon after this finding in Cyprus from June 2016, it was also reported from Cyprus in Larnaca Bay in November 2016 by Gerovasileiou et al. (2017).

Bryozoa
Family: Candidae
***Tricellaria inopinata*** D’Hondt & Occhipinti-Ambrogi, 1985

**Potential native origin:** Indo-Pacific Ocean.

**Distribution:** It is considered a NIS in New Zealand and cryptogenic elsewhere in the Pacific, from Japan to Taiwan, Australia and the Northeast Pacific (Dyrynda et al., 2000). It was also reported from the Northeast Atlantic coasts of Great Britain, Ireland, Belgium, France, the Netherlands, Spain, Portugal and Germany (Dyrynda et al., 2000; Arenas et al., 2006; Cook et al., 2013). This species has also been transported via aquaculture and in association with marine debris stemming from the 2011 Japanese tsunami which landed in Oregon (Calder et al., 2014).

In the Mediterranean, *Tricellaria inopinata* was first described in the Lagoon of Venice in 1982 (D’Hondt & Occhipinti-Ambrogi, 1985) and is considered a NIS in the Mediterranean Sea because the genus *Tricellaria*, typical of the Indo-Pacific Ocean, was previously absent from the basin. After its initial Venetian record, it was reported from Tunisia (Ben Souissi, Ben Salem & Zaouali, 2006), and from several other Italian localities (Lodola, Savini & Occhipinti-Ambrogi, 2012; Ferrario et al., 2017).

**New records:** This finding represents new country records for France (#3 and #5: Fig. S2A) and Greece (#24).

**Boat-hull records:** Found on boat-hulls moored in Italy (#14), France (#3 and #5), and Turkey (#27).

**Notes:** In Europe, it was found on various types of artificial substrates, e.g., boat-hulls, ropes, docks and also natural substrates (Dyrynda et al., 2000; De Blauwe & Faasse, 2001). Generally, *Tricellaria inopinata* is known to establish successfully in marinas lacking strong freshwater inputs (Occhipinti-Ambrogi, 1991; Johnson, Winston & Woollacott, 2012; Cook et al., 2013). If it establishes from boat to marina in Turkey, it would then present a new country record.

Family: Hippopodinidae
***Hippopodina* sp. A**

**Potential native origin:** Indo-Pacific Ocean.

**Distribution:** The species *Hippopodina feegeensis* (Busk, 1884) from the Indo-Pacific and the Red Sea, was reported as NIS in the Eastern Mediterranean Sea (Powell, 1969; Morri et al., 1999; Corsini-Foka et al., 2015). However, Tilbrook (1999) had observed strong morphological variations within *Hippopodina feegeensis* colonies from different geographical regions, and some species were later designated to be new species (Tilbrook, 2006). Particularly, Tilbrook (2006) recognised that the true *Hippopodina feegeensis* is restricted to the Philippines Islands, South China Sea and Australia, while two other *Hippopodina* spp. were left undescribed (named as *Hippopodina* “*feegeensis*,” Holothuria Bank and *Hippopodina* “*feegeensis*,” Ethiopia (*sic*) in Tilbrook, 2006). The material presented here is most likely conspecific with the still undescribed *Hippopodina* sp. collected by Tilbrook (2006) from Massawa Harbor, Erythraea (K. J. Tilbrook, 2017, personal communication), and is indicated here as *Hippopodina* sp. A.

**New records:** This study presents a new country record for Turkey (#32). It was also found in Rhodes, Greece (#26: Fig. S2B). Recently, Corsini-Foka et al. (2015) recorded *Hippopodina feegeensis* from Mandraki Harbor in Rhodes, in the same locality where it was also collected during this study (at the Three Windmills wall), and those specimens will likely be re-assigned to *Hipppodina* sp. A, after a more comprehensive and detailed taxonomic comparison is undertaken.

**Boat-hull records:** Found on two boat-hulls moored in Turkey (#32: Fig. S2C).

**Notes:** This species is morphologically similar to *Hippopodina feegeensis*, with only a few varying characters (see Supplementary Data). Further morphological and genetic comparisons are necessitated to compare the Mediterranean specimens thus far identified as *Hippopodina feegeensis* (Powell, 1969; Morri et al., 1999; Corsini-Foka et al., 2015) with samples from the Red Sea, which will then lead to a proper taxonomic description for these *Hippopodina* samples.

Family: Lepraliellidae
***Celleporaria brunnea*** (Hincks, 1884)

**Native origin:** Northeast Pacific Ocean.

**Distribution:** It is widely distributed in the Pacific Ocean (British Columbia, Ecuador, Gulf of California, Hawaiin Islands, Korea and Panama Canal: see Soule, Soule & Chaney, 1995; Seo & Min, 2009). Recorded as a NIS along the North-eastern Atlantic (Portugal and France: Canning-Clode, Souto & McCann, 2013; Harmelin, 2014) and Mediterranean Sea (from Croatia, Italy, Lebanon, Turkey: Koçak, 2007; Harmelin, Bitar & Zibrowius, 2009; Harmelin, 2014; Lezzi, Pierri & Cardone, 2015; Lodola, Ferrario & Occhipinti-Ambrogi, 2015; Ferrario et al., 2016; Marić et al., 2016).

**New records:** These findings represent first country records for Spain (#1), France (#4, #5: Figs. S2D–S2F, #6, #8), Malta (#22 and #23), and Greece (#24). In Turkey, *Celleporaria brunnea* was previously found in Izmir Bay by Koçak (2007), and during this study, three additional localities are added to its previously known Turkish distribution (#28, #30 and #31), illustrating its wider expansion along the Turkish south-western and southern coasts. During this study, it was also present all around Sicily (#15, #16, #17, #19, #20, #21).

**Boat-hull records:** Found on boat-hulls moored in France (#3, #7, #9, #10) and Greece (#25, #26), but was not found from the artificial substrates of those same marinas.

**Notes:** Many species of the genus *Celleporaria* are tolerant and opportunistic, and may exhibit invasive attributes (Dunstan & Johnson, 2004). *Celleporaria brunnea* was reported as a fouling organism from different substrates, both natural and artificial (Koçak, 2007; Canning-Clode, Souto & McCann, 2013; Lezzi, Pierri & Cardone, 2015). Furthermore, it can be easily spread via hull-fouling, but its introduction via the aquaculture trade cannot be ruled out, as some of the Mediterranean findings refer to sites in close proximity to shellfish farms (Lezzi, Pierri & Cardone, 2015; Lodola, Ferrario & Occhipinti-Ambrogi, 2015).

Family: Lepraliellidae
***Celleporaria vermiformis*** (Waters, 1909)

**Native origin:** Red Sea.

**Distribution:** Apart from the Red Sea, its distribution is not well known (Vine, 1986; Ostrovsky et al., 2011). However, it has recently been found in the Gulf of Oman (Dobretsov, 2015). Its first and only Mediterranean record (prior to our new records listed below) is from Tripoli, Lebanon (Harmelin, 2014).

**New records:** This study presents new country records for both Greece (#24, #25, #26) and Cyprus (#33: Figs. S2G–S2I, and #34).

**Boat-hull records:** Found on boat-hulls moored in Greece (#25 and #26), and Cyprus (#33 and #34).

**Notes:** Since *Celleporaria vermiformis* was previously recorded from only a single record from a single site in Lebanon, it was not previously considered as an established species (Harmelin, 2014). However, these five new locality records presented here now qualify it as an established NIS in the Mediterranean, and signifies its likely spreading in the eastern portion of the basin.

Family: Smittinidae
***Parasmittina egyptiaca*** (Waters, 1909)

**Native origin:** Red Sea and Indo-Pacific Ocean.

**Distribution:** *Parasmittina egyptiaca* was reported along the Suez Canal (Hastings, 1927; Harmelin, Bitar & Zibrowius, 2009), in the Red Sea (Ostrovsky et al., 2011), and from the Indo-Pacific region (Menon, 1972). In the Mediterranean Sea, it has only been reported from Lebanon (Harmelin, Bitar & Zibrowius, 2009) and Israel (Sokolover, Taylor & Ilan, 2016).

**New records:** This finding represents two new country records for Turkey (#32) and Cyprus (#33).

**Boat-hull records:** Found on boat-hulls moored in Greece (#25: Figs. S3A and S3B), and Italy (#21). The Italian finding presents a new Central Mediterranean record for this species.

**Notes:** In our samples, *Parasmittina egyptiaca* was mostly found growing on *Amphibalanus amphitrite* (Darwin, 1854) specimens and oysters. The captain of the boat hosting this species in Italy explained that his home marina was Finike, Turkey (#32), and he had just recently travelled from there, through Greece to Sicily. Interestingly, one could expect many similar examples of new country records for Greece as several dozens of liveaboard recreational sailboats that used to winter in the Finike marina in Turkey explained to the first author that since 2014, many had collectively relocated their vessels to now winter in Agios Nikolaos, Crete (#25). Despite thorough sampling procedures in Agios Nikolaos, this species was not found present in the marina.

Family: Vesiculariidae
***Amathia verticillata*** (Delle Chiaje, 1822)

**Native origin:** Caribbean Sea.

**Distribution:** It has a cosmopolitan distribution from tropical to subtropical regions in the Atlantic and Indo-Pacific Oceans, the Mediterranean Sea and Macaronesia (Amat & Tempera, 2009; Wirtz & Canning-Clode, 2009; Minchin, 2012; Ferrario, Marchini & Lodola, 2014; Marchini, Ferrario & Minchin, 2015).

In the Mediterranean, it was first recorded in the Gulf of Naples (Delle Chiaje, 1822) and is well-known from the following countries: Algeria, Croatia, Egypt, France, Greece, Israel, Spain, Syria and Tunisia (Marchini, Ferrario & Minchin, 2015).

**New records:** This finding represents new country records for Malta (#22, #23), Turkey (#28 and #30: Fig. S3E) and Cyprus (#34). During this study, it was also found in Spain (#2), France (#4, #5, #6, #11), the Tyrrhenian coast of Italy (#12 and #14), the Ionian Sea (around Sicily, #15–21), and Greece (#24 and #26).

**Boat-hull records:** Found on boat-hulls moored in France (#10), Italy (#12: Fig. S3F, #13, #14, #15, #17, #20, #21), Malta (#22, #23), Greece (#24–26), Turkey (#28, #30, #31), and Cyprus (#34).

**Notes:** It was recently confirmed to originate from the Caribbean (see Galil & Gevili, 2014). Due to its rapid growth rate, it can pose ecological and economic impacts by forming extensive and resistant colonies on many types of artificial substrates (Ferrario et al., 2016), and can also facilitate introductions of additional fouling species (Marchini, Ferrario & Minchin, 2015), such as *Caprella scaura*, which was found to be intertwined with it in large abundances in La Grand-Motte, France when we sampled there.

Family: Watersiporidae
***Watersipora arcuata*** Banta, 1969

**Potential native origin:** Tropical Eastern Pacific.

**Distribution:** It is a widespread species distributed from the tropical Pacific such as the Mexican Pacific coast, California and Hawaii, extending down to Australasia (Wisley, 1958; Skerman, 1960; Banta, 1969; Coles, DeFelice & Eldredge, 1999). In the Mediterranean, it had only been reported from Porto Santa Margherita Ligure in NW Italy and Porto Rotondo Marina in Sardinia (Ferrario et al., 2015, 2017).

**New records:** This finding represents new country records for Spain (#1 and #2), Malta (#22: Fig. S3I) and Turkey (#28 and #32). This also represents an additional Italian locality record for Sicily (#18: Figs. S3G and S3H, and #20). Therefore, this study shows this species is now present in all regions of the Mediterranean, presenting here two new subregional records for the Western and Eastern Mediterranean.

**Boat-hull records:** Found on a boat-hull moored in France (#7), but not found from the marina substrate.

**Notes:** In this study, *W. arcuata* was especially abundant in Siracusa, Sicily. The captain of the boat in Cogolin, France (#7) hosting this species had recently travelled from Barcelona, where it was also found in the marina from this study. If it does establish in France, it would then present a new country record.

Crustacea
Cirripedia
Family: Balanidae
***Amphibalanus improvisus*** (Darwin, 1854)

**Potential native origin:** Western Atlantic Ocean.

**Distribution:** It is considered NIS in the Pacific Northwest, and is also present in the Sea of Japan, New Zealand and northern Europe (Foster & Willan, 1979; Zullo, 1979; Furman, 1989; Iwasaki, 2006).

In the Mediterranean region, it was first reported from the Black Sea in 1844 (Gomoiu et al., 2002). Next, it was found in the Bosphorus Strait, Turkey (Neu, 1935), which connects the Black Sea to the Aegean Sea. By the late 1940s it was also reported in Barcelona (Spain), Catania (Italy), and Alexandria and Abukir (Egypt; Kolosvary, 1949).

**Marina records:** This finding represents a new locality record for Italy (#12). It was also found in Turkey (#27).

**Boat-hull records:** Found on boat-hulls moored in France (#5: Figs. S4A and S4B) and Turkey (#28).

**Notes:** If *Amphibalanus improvisus* happens to establish in Port Camargue marina, this would then present a new country record for France. The captain of a boat hosting *Amphibalanus improvisus* in Port Camargue (France #5) had recently travelled from Barcelona (where it was recorded long ago), as well as the Balearic Islands. Another captain from Port Camargue also hosting this species had recently only travelled to the Balearic Islands, so it is likely that *Amphibalanus improvisus* is present there. The captain hosting this species from Bodrum, Turkey (#28) had recently travelled to Istanbul, where this species has long been present, in addition to travelling through Italy and Greece.

***Balanus trigonus*** Darwin, 1854

**Native origin:** Indo-Pacific.

**Distribution:** It was first described from the Pacific Ocean (Darwin, 1854), and has a wide Indo-Pacific distribution extending to the Red Sea. It is considered NIS in the Atlantic Ocean and Mediterranean Sea, its first Atlantic record coming from Brazil in the 1860s (Zullo, 1992). It was introduced to the Atlantic coast of North America around the 1950s to the 1960s (Moore & McPherson, 1963; Gittings, 1985), and has also been reported from the Eastern Atlantic from the Azores to South Africa.

Its first Mediterranean record was from the Gulf of Catania, Italy in 1927 (Patane, 1927). It was abundant in the Italian Tyrrhenian, Ionian and Adriatic Seas in the 1960s (Relini, 1968). It is also reported from Egypt (Ghobashy, 1976), Lebanon (Bitar & Kouli-Bitar, 2001), Turkey (Koçak, Ergen & Çinar, 1999), Greece (Koukouras & Matsa, 1998), Croatia (Igić, 2007) and Slovenia (Mavrič et al., 2010).

**Boat-hull records:** Found on boat-hulls moored in Italy (#14 and #15), Turkey (#32), Greece (#24 and #26), and Cyprus (#33: Figs. S4C and S4D).

**Notes:** Although reported on boat-hulls in north-western Europe, it has not established in that region (Hayward & Ryland, 1995). Relini (1968) questioned a lack of other Mediterranean records for this species despite its earlier abundance in the Italian Ionian, Tyrrhenian and Adriatic Seas. In Cyprus, *Balanus trigonus* has not yet been reported for the country, and the boat captain in Cyprus hosting this species explained that he had just travelled along Turkey’s Mediterranean coast and also through Rhodes, Greece since his last hull-cleaning. If this species establishes itself in Karpaz Marina, Cyprus, where it was found on boats, it would then present a new country record for Cyprus. This species can also be transported via both the aquaculture or “Live Fish Food Trade” pathways due to its custom of gluing itself onto other marine species such as shellfish and crabs (Zullo, 1992).

Decapoda
Family: Portunidae
***Charybdis (Gonioinfradens) paucidentatus*** (A. Milne-Edwards, 1861)

**Native origin:** Indo-Pacific.

**Distribution:** This species has a wide Indo-Pacific distribution, including the Red Sea, eastern Africa, Australia, New Caledonia, Japan (Poupin, 1994, 1996; Apel & Spiridinov, 1998; Apel, 2001), Madagascar (Crosnier, 1962), the Persian Gulf (Naderloo & Sari, 2007) and Hawaii (Davie, 1998).

Its first Mediterranean record was in Turkey in 2009 from the Kaş-Kekova specially protected area from the Turkish Levantine coast (Karhan & Yokeş, 2012). A 2010 record from Rhodes, Greece provided the second Mediterranean record (Corsini-Foka et al., 2010), which is only about 140 km from Kaş, Turkey.

**New records:** This finding represents a new country record for Cyprus (#34: Fig. S5A).

**Notes:** It may have been introduced to the Eastern Mediterranean via ballast water (Corsini-Foka et al., 2010).

***Dyspanopeus sayi*** (Smith, 1969)

**Native origin:** Western Atlantic, from Canada to Florida.

**Distribution:** It spread from the Western Atlantic to the Northeastern Atlantic and also to the North Sea: Great Britain, France and Netherlands (Ingle, 1980; Clark, 1986). Its first Mediterranean record was from the Lagoon of Venice in 1991 (Froglia & Speranza, 1993), then next a little south in the Adriatic Sea in the Po River Delta (Turolla, 1999). In 2009, it was found in a Romanian harbor in the Black Sea (Micu, Niţă & Todorova, 2010), and in 2010 from the Ebro Delta of the Iberian Peninsula, providing the first Western Mediterranean record (Schubart, Guerao & Abelló, 2012). In 2011, it was collected from the central-southern Adriatic Sea lagoon of Varano (Ungaro, Pastorelli & Di Festa, 2012), and in 2011 it was reported in Mar Piccolo, Gulf of Taranto (Ionian Sea, Kapiris et al., 2014) another known hotspot for NIS, and then in Lago Fusaro (a brackish lagoon north of Naples), where it was the most abundant crab (Thessalou-Legaki et al., 2012).

**New records:** This finding represents a first subregional record for the Eastern Mediterranean and additionally a new country record for Greece (#24: Figs. S5B and S5C). It was also found in Sicily (#18) from this study.

**Notes:** Its first Mediterranean record from Venice is hypothesized to have arrived either via the ballast water or aquaculture vector (Froglia & Speranza, 1993).

Peracarida—Amphipoda
Family: Ampithoidae
***Ampithoe bizseli*** Özaydinli & Coleman, 2012

**Potential native origin:** Red Sea and Indian Ocean.

**Distribution:** To date, the species has only been reported from Tanzania and Turkey (Izmir Bay, Özaydinli & Coleman, 2012). Its distribution may be much wider than currently known, but this species could have been misidentified as *Ampithoe ramondi* Audouin, 1826, following Schellenberg’s (1928) record of “*Ampithoe ramondi*” (see notes below).

**New records:** This finding represents a new country record for Cyprus (#33 and #34: Figs. S6A and S6B).

**Boat hull records:** Found on boat-hulls moored in Cyprus (#33 and #34).

**Notes:** According to Özaydinli & Coleman (2012), specimens from Tanzania identified as *Ampithoe ramondi* by Schellenberg (1928) display ischium lobes identical to *Ampithoe bizseli*. For this reason, the native origin of *Ampithoe bizseli* is hypothesized to be the Indian Ocean, from where it could have been transferred to the Mediterranean via hull-fouling. Its current presence in two marinas and also on boat-hulls in those marinas supports the hypothesis of biofouling as a vector for its wider spread.

Family: Aoridae
***Aoroides longimerus*** Ren & Zheng, 1996

**Native origin:** Northwest Pacific Ocean.

**Distribution:** It has been reported from Daya Bay, China (Ren & Zheng, 1996) and from Osaka and Wakayama, Japan (Ariyama, 2004). It has also been recorded from the Northeastern French Atlantic coast where it is considered NIS (Gouillieux et al., 2016).

**New records:** This finding represents a new Mediterranean record (#5: Figs. S6C and S6D), and a new regional record for the French Mediterranean.

**Boat-hull records:** Found on boat-hulls in France (#5).

**Notes:** Port Camargue, France, is situated in close proximity to Thau lagoon, the most important Mediterranean locality for aquaculture farming of Japanese oysters (Boudouresque et al., 2011). This information and our new record from boat-hulls suggests that both aquaculture and shipping are possible vectors of introduction, similarly to what has been indicated for the French Atlantic record (Gouillieux et al., 2016).

***Bemlos leptocheirus*** (Walker, 1909)

**Native origin:** Red Sea, Indian Ocean.

**Distribution:** Aside from early records from its native region: Kenya, Tanzania, South Africa, Suez Canal (Walker, 1909; Schellenberg, 1928; Sivaprakasam, 1968; Myers, 1975), its first and only Mediterranean record was from Egyptian coast from Port Said, Alexandria, and Abu Kir in the early 20th century (Schellenberg, 1928; Bellan-Santini et al., 1998). However, it was considered to be as absent from the Mediterranean, as it had not been reported since (Zenetos et al., 2017).

**New records:** This finding represents a new country record for Greece (#24: Figs. S6E and S6F, and #25), and confirms its presence and reappearance in the Eastern Mediterranean.

**Notes:** Previous findings of *Bemlos leptocheirus* in the Suez Canal and from the Egyptian Mediterranean coast, near the canals entrance, suggest it to have a “Lessepsian migrant” vector status (Bellan-Santini et al., 1998), especially since it was also recorded from buoys and boats (Schellenberg, 1928). Our findings support that it should rather be assigned to the “biofouling or hull-fouling” vector.

Family: Ischyroceridae
***Ericthonius* cf. *pugnax*** (Dana 1852)

**Potential native origin:** Indonesia.

**Distribution:** *Ericthonius pugnax* has a wide Indo-Pacific distribution including Australia (Great Barrier Reef, New Caledonia and New South Wales), Papua New Guinea, Singapore, Japan, Korea, Malaysia, India, Sri Lanka, Madagascar and Mauritius (Marchini & Cardeccia, 2017 and references therein). It was reported from New Zealand as a NIS (Ahyong & Wilkens, 2011). Records of *Ericthonius pugnax* from South Africa on mussel rafts (Milne & Griffiths, 2013) may also represent an introduction event. In the Mediterranean Sea, a record of *Ericthonius dydimos* from the Adriatic Sea (Krapp-Schickel, 2013) may refer to this species.

**New records:** This finding represents a new subregional record for the Western Mediterranean and a new country record for France (#5: Figs. S6G and S6H).

**Boat-hull records:** Found on boat-hulls moored in France (#3 and #5).

**Notes:** An *Ericthonius* species strikingly similar to *Ericthonius pugnax* was described by Krapp-Schickel (2013) from the Lagoon of Venice (and to date, has not been reported from other localities): *Ericthonius didymus*. The latter presents a strongly posteriorly lobate pereopod 5 basis, identical to *Ericthonius pugnax*. Krapp-Schickel (2013) justifies the establishment of the new species *Ericthonius didymus* on the basis of differences in pereopod 5 postero-distal lobe (in *Ericthonius didymus* only visible in adult males; in *Ericthonius pugnax*, visible in both adult and juvenile males), in shape of gnathopod 2 carpus (bearing two teeth in *Ericthonius didymus*, versus only one tooth in *Ericthonius pugnax*), shape of pereopods 3 and 4 basis, as well as body size. However, a re-examination of *Ericthonius* material collected in 2012 from the Lagoon of Venice (A. Marchini, 2012, private collection), together with a cross-comparison of descriptions and drawings of both *Ericthonius didymus*, provided by Krapp-Schickel (2013), and *Ericthonius pugnax*, provided by Moore (1988), Just (2009) and Azman & Othman (2013), shows that the differences pointed out by Krapp-Schickel (2013) may not support the separation between the two species. With regards to gnathopod 2 carpus, Azman & Othman (2013) showed that the number of teeth in male *Ericthonius pugnax* varies with maturity. Furthermore, we observed some males from Venice having a single-toothed gnathopod 2 carpus, consistent with the description of *Ericthonius pugnax* hyperadult males of Moore (1988). The basis of pereopods 3, 4 is bottle-shaped, and distally expanded in both species. Furthermore, body length is largely variable (*Ericthonius didymus* described from Venice is 4.5 mm; *Ericthonius pugnax* described from Australia by Moore (1988) and Just (2009) is 3.0–3.7 mm, from Malaysia by Azman & Othman (2013) is 3.8 mm, from Japan by Nagata (1965) is up to 7.5 mm.

Therefore, we hereby suggest that the “endemic” *Ericthonius didymus* in Venice may be an introduced population of the Indo-Pacific *Ericthonius pugnax*, and therefore may be a pseudoindigenous species (see definitions). However, it is also possible that the global populations of *Ericthonius* with a posteriorly lobated pereopod 5 basis represent a complex of cryptic species. We consider that in this case the hypothesis of the valid introduced status is supported by the following facts:

1. *Ericthonius pugnax* has a notably wide distribution in the Indo-Pacific region, which supports a human-mediated dispersal hypothesis, and is already known as a NIS from New Zealand (and possibly, South Africa);
2. In the Lagoon of Venice, it has developed populations with high densities (A. Marchini, personal observation), which is consistent with “invasive” behaviour; and
3. The Lagoon of Venice is a well-known hotspot of introductions, where over 70 NIS have already been recorded, many with Pacific/Indo-Pacific origins, which were introduced to Venice *via* oyster imports (Marchini, Galil & Occhipinti, 2015). The present records from Cap d’Agde and Port Camargue are both nearby another popular hotspot for oyster introductions, the Thau lagoon (Boudouresque et al., 2011). This further supports the hypothesis of introduction from the Indo-Pacific region, with aquaculture being the main pathway of primary introduction.

Family: Stenothoidae
***Stenothoe georgiana*** Bynum & Fox 1977

**Native origin:** Western Atlantic.

**Distribution:** Its first record outside its native range was reported just recently in 2010, in association with fouling communities of offshore fish farms (about 10 km from shore) in Alicante and Murcia, Spain (Fernández-González & Sánchez-Jerez, 2017). Its subsequent Mediterranean records were from the Ligurian Sea and from Sardinia, Italy (Ferrario et al., 2017).

**New records:** These findings represent new country records for France (#5) and Malta (#23). This study increases its known Italian distribution by incorporating Sicily (#14: Figs. S6I and S6J, #15, #18, #21). The Maltese and Sicilian findings from this study represent a new Central Mediterranean subregional record.

**Boat-hull records:** Found on boat-hulls moored in France (#3, and #10), and Italy (#14, #15, #17, and #21).

**Notes:** Since this species has only very recently been reported in the Mediterranean, we hypothesize that it may have gone previously overlooked, since it is already present in at least four countries. It may soon establish in Cap d’Agde Marina or Port Vauban, Antibes, and this should serve as a warning for future monitoring of those marinas. This study demonstrates that this species is likely polyvectic (see definitions): in addition to its likely transfer via aquaculture (Fernández-González & Sánchez-Jerez, 2017), recreational boating is also facilitating its spread.

Peracarida—Isopoda
Family: Anthuridae
***Mesanthura* cf. *romulea*** Poore & Lew-Ton, 1986

**Potential native origin:** Tropical to sub-tropical southern seas.

**Distribution:** *Mesanthura* specimens belonging to the same species and sharing major diagnostic characters with *Mesanthura romulea* described from Australia (Poore & Lew-Ton, 1986) were subsequently (2000) collected from the harbors of Salerno and Taranto (Italy), where they were well established (Lorenti, Dappiano & Gambi, 2009), and also from Ischia Island (Kroeker et al., 2011). More recently, this species has been reported by Ferrario et al. (2017) from marinas in Northern Italy (Liguria).

**New records:** This finding represents new country records for Spain (#1: Fig. S7A), Malta (#22: Fig. S7A), Greece (#26) and Cyprus (#33 and #34), the latter two records also confirming the presence of *Mesanthura* cf. *romulea* in the Eastern Mediterranean. From this study, we additionally report specimens from Italy (#14, #15, #16: Fig. S7A, and #18).

**Notes:** The earliest mention of the presence of the genus *Mesanthura* in the Mediterranean region was from Lake Burullus, Egypt (Samaan, Ghobashy & Aboul Ezz, 1989); however, the record was not supported with taxonomic details and needs confirmation. Castelló (2017) recently described a new *Mesanthura* species from both the Lebanese coast and Cyprus (*Mesanthura pacoi,* Castelló, 2017), whose females vary from those of the present species in the dorsal colour pattern and in other subtle morphological features. As mentioned above, the species found by Lorenti, Dappiano & Gambi (2009) and reported here is comparable and most probably conspecific (G. Poore, 2017, personal communication) with *Mesanthura romulea* described by Poore & Lew-Ton (1986), which is based only on two specimens collected from Sydney Harbor and Port Stephens, New South Wales, Australia. No other records of this species have been published.

The fact that the extant description of the Australian *Mesanthura romulea* lacks a number of taxonomic characters and is based on only two specimens prevents from determining if features observed in all Mediterranean specimens lie within the natural range of morphological variation of the species, or allow for the determination of a different species.

As long as these cases of taxonomic identity are unsolved, and no new material of *Mesanthura romulea* is found from its putative native range, the origin of populations occurring in the Mediterranean remains obscure. However, the Mediterranean finding of the present species of *Mesanthura* shows strong indications of a human-mediated introduction. Following Chapman & Carlton’s (1991) criteria, the lack of previous records of the genus *Mesanthura* on a basin scale (except for the recent discovery of *Mesanthura pacoi* from the Levantine Sea), the mentioned occurrences from confined areas such as lagoons and harbors, the notably poor capabilities of active or passive spreading by natural means of the genus, and its likely exotic evolutionary origin, cumulatively support the hypothesis of a human-mediated introduction.

Family: Janiridae
***Ianiropsis serricaudis*** Gurjanova, 1936

**Native origin:** Sea of Okhotsk to the Sea of Japan.

**Distribution:** In addition to its native range, it has been reported from the Northeastern Pacific (from Puget Sound to Monterey Bay), the Northwestern Atlantic (from Maine to New Jersey) and the Eastern Atlantic and North Sea (England and the Netherlands) (Hobbs et al., 2015).

Its first Mediterranean record was in 2012 from the Lagoon of Venice (Marchini, Ferrario & Occhipinti-Ambrogi, 2016a), and soon after from Olbia, Sardinia in 2014 (Marchini, Ferrario & Occhipinti-Ambrogi, 2016b).

**New records:** This finding represents a new country record for France (#3 and #5: Fig. S7B).

**Notes:** In North America, this species is now known as a common fouling species. It was hypothesized that this species is likely more established along North America and the European coasts than what is known, but may go undetected due to its minuscule size (<3 mm) and the taxonomic complexity of the genus (Hobbs et al., 2015). All the Mediterranean findings (Venice, Olbia, Port Camargue) refer to sites in close proximity to aquaculture sites.

Family: Paranthuridae
***Paranthura japonica*** Richardson 1909

**Native origin:** Northwest Pacific Ocean.

**Distribution:** It was first reported from Muroran, northern Japan and from eastern Russia (Nunomura, 1977). It was reported as a NIS for San Francisco Bay in 1993, then from southern California in 2000 (Cohen & Carlton, 1995; Cohen, 2005). Between 2007 and 2010 it was first recorded in European waters from the Bay of Biscay, France, most likely via the aquaculture vector (Lavesque et al., 2013).

Its first Mediterranean records occurred only recently; between 2010 and 2012 it was found in numerous localities around Italy: the Lagoon of Venice, La Spezia and Olbia harbors (Marchini et al., 2014), and Taranto (Lorenti et al., 2015). Next, it was found in La Grande-Motte, France (Marchini, Ferrario & Minchin, 2015) and then in Tunisia and Greece (Tempesti, Langeneck & Castelli, 2016).

**New records:** These findings represent new country records for Spain (#1: Fig. S7C, and #2) and Malta (#23). Furthermore, *Paranthura japonica* was found in countries where it was already reported from, extending its known distribution to new localities in France (#3, #4 and #9), Italy (#12, #13, #16–21: (#21) Fig. S7C), and Greece (#24 and #26). These new Sicilian records (#16–21), and Maltese record (#23) show it is already well-established in the Central Mediterranean.

**Boat-hull records:** Found on boat-hulls moored in France (#3, #5, #9 and #10), Italy (#12, #17, #20 and #21) and Greece (#24).

**Notes:** The current findings dramatically increase the known distribution of *Paranthura japonica*, revealing it as one of the most widespread NIS in the Mediterranean Sea. While the initial findings of *Paranthura japonica* had suggested an association with aquaculture transfers, these new records show that it most likely is a polyvectic species (see definitions) species, which complicates the possibility of reconstructing its invasion trajectory.

Family: Sphaeromatidae
***Cymodoce* aff. *fuscina*** Schotte & Kensley, 2005

**Native origin:** Persian Gulf.

**Distribution:** *Cymodoce fuscina* was first described in 2005 from seagrass beds in Saudi Arabia, the Persian Gulf by Schotte & Kensley (2005). Until now, this isopod had not been reported outside the Persian Gulf.

**New records:** This finding represents a new record for the Mediterranean basin, and a new country record for Greece (#24: Figs. S7D and S7E).

**Boat-hull records:** Found on boat-hulls moored in Greece (#24 and #25).

**Notes:** Our specimens show very strong affinity to *Cymodoce fuscina* from the Persian Gulf (V. Khalaji-Pirbalouty, 2016, personal communication), and they certainly differ from all other known *Cymodoce* species reported in the Mediterranean Sea in several characters of the pleotelsonic region, while also being similar to other species described from the Western Indian Ocean (Khalaji-Pirbalouty & Raupach, 2014). Its association with marina structures and hull-fouling further supports the hypothesis of a human-mediated introduction, possibly from boats travelling from the Red Sea through the Suez Canal. However, slight differences between our material and the original description of *Cymodoce fuscina* by Schotte & Kensley (2005) should be noted, for example the pleotelsonic apex of *Cymodoce fuscina* has the three apical lobes subequal in length and rounded apically, while in our material the central lobe is slightly longer than the lateral ones, and ends in a tiny bifid spike. We stress the fact that not all Indo-Pacific species within this genus may be known (many new species have been described in the recent decade), and a complex of species is also a possibility. Therefore, we recommend that genetic analyses should be undertaken to compare the Mediterranean material with specimens from the native range, to confirm the identity of these samples from Heraklion, Greece.

***Paracerceis sculpta*** (Holmes 1904)

**Native origin:** California.

**Distribution:** This is a widely distributed species naturally found along the North American Pacific coast from California to Mexico, and has also been reported from Hawaii, Hong Kong, Australia, Brazil and the Azores (Marchini et al., in press and references therein).

In the Mediterranean Sea, it was first reported from the Lake of Tunis, Tunisia (Rezig, 1978); and next from several Italian localities (Forniz & Sconfietti, 1983; Forniz & Maggiore, 1985; Savini et al., 2006; Ferrario et al., 2017), and the Strait of Gibraltar (Castelló & Carballo, 2001). Most recently, it was reported in Thermaikos and Toroneos Gulf in Greece (Katsanevakis et al., 2014) and La Grande-Motte in France (Marchini, Ferrario & Minchin, 2015).

**New records:** This finding represents new country records for both Malta (#22: Fig. S7F, and #23) and Cyprus (#34). It was also found in France (#4), Greece (#24 and #26) and Italy (#13, #15–21).

**Boat-hull records:** Found on boat-hulls from Sicily (#17, #20 and #21), Greece (#24) and Turkey (#31).

**Notes:** This species has often been reported from marinas, indicating that recreational boating plays an important role in the spread of this global invader. In Fethiye (#31), it was found on a boat-hull but not in the marina and so far was unknown from Turkey; When interviewed, the boat captain hosting this species explained he had just travelled from Rhodes (#26), where it was found in the marina. Attention should be paid to see if it spreads to the marina in Fethiye, Turkey, where it would then constitute a new country record.

***Paradella dianae*** (Menzies, 1962)

**Native origin:** Eastern Pacific Ocean.

**Distribution:** The first description of this species was from the Bay of San Quintin, Baja California (Menzies, 1962).

Its first Mediterranean record was from Civitavecchia, Italy (Forniz & Maggiore, 1985), followed by a series of findings in Egypt (Atta, 1991), Spain (Castelló & Carballo, 2001), Turkey (Çinar et al., 2006), Cyprus (Kırkım et al., 2010), Libya (Zgozi, Haddoud & Rough, 2002) and Sardinia, Italy (Ferrario et al., 2017).

**New records:** This finding represents a new locality record for Sicily, Italy (#15: Fig. S7G), and an additional record for Turkey from the same locality (Fethiye) it had previously been reported in (#31).

**Boat-hull records:** Found on boat-hulls moored in Greece (#24), and Italy (#20).

**Notes:** This species has not yet been reported in Greece, so this finding on a boat-hull in Heraklion, Crete, which had only travelled through Greek islands since its last cleaning alludes to its presence in Greek waters. Interestingly, the boat-hull it was found on in Sicily had just travelled from Fethiye, Turkey, where it is known from. It is assumed that this sphaeromatid isopod arrived to the Mediterranean via hull-fouling on vessels from the Northeast Pacific, its alleged original native range (Galil, Occhipinti-Ambrogi & Gollasch, 2008).

***Sphaeroma walkeri*** Stebbing 1905

**Native origin:** Indian Ocean.

**Distribution:** This species is commonly found in intertidal fouling communities and has been widely reported from ports in warm and warm-temperate waters worldwide, including the Pacific coast of North America (Carlton & Iverson, 1981).

Its first Mediterranean record is from Port Said, Egypt in 1924, where it was found on boat-hulls (Omer-Cooper, 1927). Half a century later (in 1977), it was reported from Toulon, France (Zibrowius, 1992), then from Turkey (Kocatas, 1978), and Alicante, Spain in 1981 (Jacobs, 1987). Decades later it was found once again on boat-hulls in Haifa Harbor, Israel (Galil, Occhipinti-Ambrogi & Gollasch, 2008), and also found to be well-established in Tunisian harbors and lagoons (Ounifi-Ben Amor, Ben Salem & Ben Souissi, 2010). In 2010 it was first spotted in Italy in the harbor of La Spezia (Lodola et al., 2012).

**New records:** This finding represents a new country record for Greece (#24: Fig. S7H). It was also found in Turkey (#31).

**Boat-hull records:** Found in Greece (#24 and #25), and Turkey (#31). This presents a new locality record for Greece (#25) in addition to the new country record presented above.

Mollusca
Family: Chamidae
***Pseudochama* cf. *corbierei*** (Jonas 1846)

**Native origin:** Red Sea, Gulf of Aqaba and Suez Canal.

**Distribution:** It is considered endemic to the Red Sea and Suez Canal (Barash & Danin, 1972). Its first Mediterranean record is from Greece (Ralli-Tzelepi, 1946), and it has also been reported from Turkey (Cachia & Mifsud, 2017). The latest record from Malta represents its first Central Mediterranean record (Cachia & Mifsud, 2017).

**New records:** One juvenile specimen was found in Italy (#20: Fig. S8F).

**Notes:** This species was formerly known as *Chama corbieri,* while *Pseudochama cornucopia* (Reeve, 1846) and *Pseudochama ruppelli* (Reeve, 1847) are both considered common synonyms. An additional record from Israel (Barash & Danin, 1972) as *Chama cornucopiae* Reeve, 1846 was excluded since the record was based on an empty shell. The present finding in Ragusa, Sicily (Italy) of a single young specimen remains dubious about its exact determination. Hence, we classify this finding as uncertain since the defining characters for this species were not yet fully developed in our juvenile specimen and suggest that the occurrence of *Pseudochama corbierei* awaits further confirmation before considering the species introduced to Italy.

Family: Mytilidae
***Arcuatula senhousia*** (Benson 1842)

**Native origin:** Siberian Peninsula to Indo-Pacific.

**Distribution:** It has been reported from Great Bitter Lake, the Suez Canal, the Red Sea, Mauritius, Zanzibar, and several Indo-Pacific and Indian Ocean countries including Thailand, Malaysia and New Caledonia (Barash & Danin, 1972).

Its first Mediterranean record was from Israel in 1960, and then from Lake Bardawil on the Egyptian Sinai Peninsula in 1982 (Barash & Danin, 1971). It was also found in Thau Lagoon, France in 1982, a popular oyster aquaculture locality (Hoenselaar & Hoenselaar, 1989) and then spread to the surrounding area including the Leucate Lagoon. Next, it was recorded in Ravenna, the Italian Adriatic coast in 1986 (Lazzari & Rinaldi, 1994). In this century, it was found in the Gulf of Olbia, Tyrrhenian Sea (Savarino & Turolla, 2000), then in 2001 it was established in the Gulf of Taranto, the Ionian Sea, from an area involving both mussel aquaculture and intense shipping (Mastrototaro, Matarrese & D’Onghia, 2003). Next, it was reported again along the Adriatic Italian coast (Solustri, Morello & Froglia, 2003), and the following year it had dense populations inside the dams of the Port of Leghorn (Livorno, Italy) (Campani et al., 2004; Margelli et al. 2004). It was also found in Tunisia (Ben Souissi et al., 2005), then, between 2006 and 2009, in Siracusa’s Porto Grande Marina, Sicily (Brancato & Reitano, 2009). In 2010, it was found in the Eastern Adriatic from the Neretva River Delta growing on serpulid tubes of the polychaete *Ficopomatus enigmaticus* (Fauvel, 1923) (Despalatović et al., 2013). In Spain, it was reported from the Ebro River Delta in 2014 (Soriano & Salgado, 2014); however, that record was based on four empty shells, therefore, its presence in Spain still awaits confirmation from live specimens.

**New records:** This finding represents the first confirmed country record for Spain (#2: Figs. S8B and S8C). It was also found in France (#5 and #9), and Sicily (#15, #16 and #21).

***Septifer cumingii*** Récluz, 1848

**Native origin:** Indo-Pacific.

**Distribution:** It is well known from the Red Sea, particularly from the Arabian coast and also from East Africa. It has also been reported from New Zealand (Maxwell, 2009), the Philippines, South China Sea and Polynesia (Huber, 2010).

Its first Mediterranean record is from Yumurtalik, Turkey in 1999 (Albayrak & Çeviker, 2001), then from Kuşadesi, Turkey (Aegean Sea) in 2000. Then a decade later, it was reported from Cyprus and the Greek Dodecanese island of Astypalaia (Zenetos, Konstantinou & Konstantinou, 2009; Zenetos et al., 2011), the Levantine coast of Turkey (Bakir et al., 2012), Saronikos Bay, Western Aegean (Zenetos et al., 2013), the Gulf of Thermaikos (Manousis & Galinou-Mitsoudi, 2014), and Lesbos Island, mid-Aegean Sea (Evagelopoulos et al., 2015). It was just reported from Paleokastritsa, Corfu (Romani et al., 2017), but only from empty shells, therefore this locality awaits verification from live specimens.

**New records:** This study presents a new locality record for Greece (Crete #25: Fig. S8A), along with its southernmost record in the Eastern Mediterranean.

**Notes:** Formerly considered a separate species, *Septifer forskali* Dunker, 1855 is now officially known as *Septifer cumingii* (Huber, 2010). From this study, it was found to be abundant both in Turkey (#29) and Cyprus (#33).

Family: Ostreidae
***Dendostrea folium sensu lato*** (Linneaus 1758)

**Native origin:** Indo-Pacific.

**Distribution:** Its first Mediterranean record is from Iskenderun Bay, Turkey in 1998 (Çeviker, 2001), then from Cyprus (Zenetos, Konstantinou & Konstantinou, 2009), and next from the Greek islands of Astypalaia, Rhodes and Kastellerizo (Karachle et al., 2016). It has also recently been reported from Panama (Lohan et al., 2015).

**New records:** This finding represents a new subregional record for the Central Mediterranean, and a new country record for Malta (#22 and #23). It was also found in Greece (#24 and #26), Turkey (#29, #30 and #32) and Cyprus (#33: Fig. S8I), where it was previously known.

**Boat-hull records:** Found on boat-hulls moored in Italy (#17), Greece (#26), Turkey (#31 and #32) and Cyprus (#33).

**Notes:** If it establishes in the marina in Italy, where it was found on a boat-hull, it would then present a new country record; the boat which was hosting *Dendostrea folium* in Italy had just returned from a long trip back from southern Turkey and the Greek Islands. *Dendostrea frons* (Linnaeus, 1758) and *Dendostrea folium* are very similar species. Huber (2010) rejects the possible presence of *Dendostrea frons* in the Mediterranean Sea, despite many reports of this species there. Based on genetic results, Crocetta et al. (2015) demonstrated that the Greek and Turkish material belongs to a single, morphologically highly variable species: *Dendostrea folium*, most likely representing a complex of species in need of revision (M. Oliverio, 2017, personal communication).

***Saccostrea* cf. *cucullata*** (Born 1778)

**Native origin:** Indo-Pacific.

**Distribution:** It is found from the Red Sea, East Africa down to South Africa including Madagascar, and West Africa up to Angola (Branch et al., 2002).

Its first Mediterranean record is from south-eastern Turkey in 1998–1999 from Erdemli, and later from Yumurtalik and Tasuçu (slightly west and east of Erdemli, respectively), where it is well-established with large populations (Çevik, Öztürk & Buzzuro, 2001), followed by a record from El-Faham, Egypt (Gofas & Zenetos, 2003). An additional record from Tunisia remains questionable (Ounifi-Ben Amor et al., 2016).

**New records:** This finding represents a possible new country record for Greece (#24). From this study, it was also found in Turkey (#31), presenting the most south-western record for the country.

**Notes:** The only specimen collected in Heraklion (Greece) was a juvenile (20 mm) and the crenulations along the margin (a key identification character) were only partially visible (Figs. S8G and S8H), but were not well developed as in matured specimens. Therefore, we regard this finding as uncertain and suggest the occurrence of *Septifer cucullata* needs further confirmation before officially presenting this as a new country record in Greece.

***Saccostrea glomerata*** (Gould 1850)

**Native origin:** Australasia.

**Distribution:** Its native distribution extends from eastern Australia to New Zealand. In the Mediterranean, it was intentionally introduced to the Adriatic Sea in 1984 for aquaculture (Cesari & Pellizzato, 1985), but has not been found there since 1990 (Mizzan, 1998), and is thus currently considered as locally extinct. In 1998 it was reported in Turkey, which was the first Eastern Mediterranean record (Çevik, Öztürk & Buzzuro, 2001), but this record is considered a case of misidentification with either *Septifer cucullata* (according to Gofas, 2011) or *Dendostrea frons* (acccording to Albayrak, 2011), so this record remains questionable.

**Boat-hull records:** This species was found on one boat-hull moored in France (#10: Figs. S8D and S8E), which had only travelled locally around the French Riviera (from Nice to Golfe-Juan) for the past 1.5 years since its last hull-painting.

**New Mediterranean records:** This finding confirms its presence in French waters and also presents a new subregional record for the Western Mediterranean.

**Notes:** This species was formerly known as *Saccostrea commercialis* (Iredale & Roughley, 1933), and is distinct from *Septifer cucullata* in terms of DNA 16S sequences (Lam & Morton, 2006; Salvi, Macali & Mariottini, 2014).

Polychaeta
Family: Serpulidae
***H. brachyacantha sensu lato*** Rioja 1941

**Potential native origin:** Mexican Pacific.

**Distribution:** Since its initial Mexican record, it has been reported globally, from Hawaii (Straughan, 1969), Brazil (Zibrowius, 1970), Micronesia (Imajima, 1982), Japan (Imajima, 1987), Venezuala (Díaz Díaz & Liñero-Arana, 2001), California (Bastida-Zavala & ten Hove, 2003) and India (Pati, Rao & Balaji, 2015).

Its first Mediterranean record was from Israel in 1933 (Ben-Eliahu, 1991), and its second from Turkey (Çinar, 2006).

**New Mediterranean records:** This finding represents new country records for both Greece (#24) and Spain (#2: Figs. S9A–S9C), the latter also presenting a new subregional record for the Western Mediterranean.

**Boat-hull records:** Found on boat-hulls moored in Greece (#24).

**Notes:** The recent paper by Sun et al. (2016) re-described *H. brachyacantha* as a complex of species, which renders the identity of the Mediterranean populations as unknown, until genetic analyses are performed and the status of the species within the complex is clarified. Consequently, the native origin of the Mediterranean populations is also unknown, and this serpulid should therefore be classified as “cryptogenic.” However, the possibility that *H. brachyacantha* is a native Mediterranean species having long escaped detection is not fully supported; it first appeared in the Mediterranean as early as in 1933 and so far has only two records in the Levantine Sea (Israel and Turkey). According to Chapman & Carlton’s (1991) criteria, these characteristics, combined with the fact that the species of *H. brachyacantha* complex are more widely distributed elsewhere (Sun et al., 2016), support a likely introduced status for the *H. brachyacantha* complex in the Mediterranean Sea.

The new records of this complex of species presented from this study in Greece and Spain demonstrate its ongoing spread, and additionally provide an important reference for future genetic analyses. Due to the uncertainty surrounding the real identity of any Mediterranean *H. brachyacantha* material, we here use the open nomenclature qualifier “*sensu lato*.”

***Hydroides dirampha*** Mörch, 1863

**Potential native origin:** Tropical Western Atlantic.

**Distribution:** Circumtropical (Bastida-Zavala & ten Hove, 2003), originally described from the US Virgin Islands (Zibrowius, 1971). It was reported in the Red Sea (Zibrowius, 1971), the Western Atlantic (Bastida-Zavala & ten Hove, 2002), the Eastern Pacific (Bastida-Zavala & ten Hove, 2003), Australia (Hayes & Sliwa, 2003; Sun et al., 2015), and Hawaii (Bastida-Zavala, 2008).

In the Mediterranean, it was first reported in Italy in 1870 as *Eupomatus lunifer* (Claparède, 1870). It has since spread all over the basin, being next reported in Spain in 1923, Egypt in 1924 (for both records: Zibrowius, 1973), Israel in 1937 (Ben-Eliahu & ten Hove, 1992), Tunisia in 1969 (Zibrowius, 1978), Lebanon in 1978 (Zibrowius & Bitar, 1981), Turkey in 2005 (Çinar, 2006) and Greece in 2014 (Corsini-Foka et al., 2015).

**New records:** This finding represents a new country record for Malta (#22 and #23: Figs. S9D and S9E).

**Boat-hull records:** Found on boat-hulls moored in marinas in France (#7), Italy (#12, #15, #17, #20, #21), Malta (#22 and #23), Greece (#24 and #25), Turkey (#31 and #32) and Cyprus (#33).

**Notes:** It is a NIS in the Mediterranean believed to be arrived by the shipping pathway from the tropical Western Atlantic (Zibrowius, 1992).

***Hydroides elegans*** (Haswell, 1883)

**Native origin:** Australasia and Indian Ocean.

**Distribution:** Circumtropical: Pacific Ocean, Caribbean, Atlantic and Northern Europe. In the Mediterranean Sea, it has been reported since the 19th century (Claparède, 1870), and has since spread to most countries in the basin (Galil & Gevili, 2014).

**New records:** This finding represents a new country record for Malta (#22). This species was found in all marinas, except for #8, #13, #14, #20, #23, #29-31, #33, #34, #35. Figs. S9F and S9G are from #18.

**Boat-hull records:** Found on boat-hulls from all marinas which had boats sampled.

**Notes:** It is considered the main fouling organism in the Mediterranean Sea (Koçak, Ergen & Çinar, 1999); our study confirms that it is the most widespread fouling species found here in terms of distribution.

***Hydroides homoceros*** Pixell, 1913

**Potential native origin:** Indo-Pacific.

**Distribution:** It was originally described from the Cape Verde Islands, in the Eastern Atlantic (Pixell, 1913). Also reported from the Red Sea, Suez Canal, Arabian Gulf, Zanzibar and Maldives (Ben-Eliahu & ten Hove, 2011).

Its first Mediterranean record was from Israel in 1955 (Ben-Eliahu, 1991), then in late 1970s from an aircraft carrier moored in Toulon, France (Ben-Eliahu & ten Hove, 2011). Next it was reported from the south-eastern Turkey (Çinar, 2006).

**Boat-hull records:** Found on boat-hulls moored in Cyprus (#33: Figs. S9H and S9I), but was not found in the same marina. The captain of one boat hosting this species in Cyprus had recently travelled to the Turkish Levantine coast, where it is known from. If it does establish in Cyprus, it would then present a new country record.

***Spirobranchus tetraceros sensu lato*** (Schmarda 1961)

**Native origin:** Indo-Pacific.

**Distribution:** First described from Australia, it has a circumtropical distribution that includes the Suez Canal, Indian Ocean, South Africa, Australia, Malaysia, Japan, China and the Caribbean (Ben-Eliahu & ten Hove, 1992; Fiege & Sun, 1999).

Its first Mediterranean record was from Lebanon in 1965 (Laubier, 1966) as *Spirobranchus giganteus coutierei* Gravier, 1908 (which is now understood as a sub-species of *Spirobranchus tetraceros*; E. Kupriyanova, 2017, personal communication), followed by Rhodes, Greece (Dumont & Werger, 1989), Abu Kir Bay, the Egyptian Mediterranean (Selim et al., 2005), and the Turkish Levantine Sea (Çinar et al., 2006).

**New records:** This finding presents a new subregional record for the Central Mediterranean and a new country record for Italy (#18: Fig. S9J). It was also found in Greece (#24).

**Notes:** *Spirobranchus tetraceros* has been treated as a complex of species since 1994 (Fiege & Sun, 1999; Ben-Eliahu & ten Hove, 2011) in need of taxonomic revision, hence, here it is referred to as *Spirobranchus tetraceros sensu lato*.

Porifera
Family: Amphoriscidae
***Paraleucilla magna*** Klautau, Monteiro & Borojevic, 2004

**Potential native origin:** Indo-Pacific and Australia.

**Distribution:** First described from the Western Atlantic in Rio de Janeiro, Brazil, and was also found from the Azores, Madeira and Portugal (Bertolino et al., 2014; Guardiola, Frotscher & Uriz, 2016).

Its first Mediterranean records were from multiple Italian localities, first in the Ionian then in the Tyrrhenian and Adriatic Seas, followed by the Ligurian Sea and Sicily (Longo et al., 2004, 2012; Longo, Mastrototaro & Corriero, 2007; Bertolino et al., 2014; Marra et al., 2016). It was also reported from multiple localites in the Costa Brava region in Spain (Guardiola, Frotscher & Uriz, 2012, 2016), as well as Malta (Zammit, Longo & Schembri, 2009), and Croatia (Cvitković et al., 2013). Its first Eastern Mediterranean record is from the Gulf of Thessaloniki, Greece, where it was first observed in 2014 in a mussel farm (Gerovasileiou et al., 2017). It has also emerged in the Sea of Marmara, Turkey in 2012 (Topaloğlu, Evcen & Çınar, 2016).

**New records:** This finding represents a new country record for Cyprus (#34), and new locality records for both Greece (#24: Fig. S10A, and #26) and Sicily. Specifically, it was present at all seven sampled Sicilian marinas (#15–21), and was also found in Malta (#23).

**Boat-hull records:** Found on boat-hulls in France (#5), Malta (#22), Greece (#26) and Cyprus (#34).

**Notes:** Prior to the recent 2004 description of *Paraleucilla magna*, this genus was only known from the Indo-Pacific region and Red Sea. As it was initially described from Rio de Janeiro in 2004, where it is considered cryptogenic (Cavalcanti et al., 2013), it was likely already present in several Mediterranean locations. For instance, the species had already been recorded (preceding its formal description) in 2001 from Mar Piccolo of Taranto (Longo et al., 2004), and according to local mussel farmers was present there as long as 20–30 years earlier (Longo, Mastrototaro & Corriero, 2007). The opportunistic behavior of *Paraleucilla magna*, with proliferation only close to either aquaculture facilities or harbours, may be the reason behind its late detection in the Mediterranean (Guardiola, Frotscher & Uriz, 2012). Moreover, as several introductions probably occurred in a short period of time, the phylogeographic signal could be weak or even lost, making the determination of the introduction pathway a challenge (Pineda, López-Legentil & Turon, 2011). Aquaculture and shipping are the most probable vectors for its recent expansion along the Western Mediterranean coast (Longo, Mastrototaro & Corriero, 2007). This study shows that *Paraleucilla magna* is now both a common and established species around Sicily and Malta. Noteworthy is the record on boat-hulls reported here from France (but not in the marina), which may represent the first step of subsequent spreading in the Western Mediterranean and recreational boating as another vector of spread.

Pycnogonida
Family: Ammotheidae
***Achelia sawayai sensu lato*** Marcus, 1940

**Native origin:** Western Atlantic.

**Distribution:** Extremely common in the tropical shallow waters of the Western Atlantic. It is distributed from Georgia to the Gulf of Mexico and throughout the Caribbean Sea to Brazil (Müller & Krapp 2009). It has also been reported in Western Africa, Madagascar and in the southern Pacific in French Polynesia, Indonesia, Fiji and Papua New Guinea, although some of these records are still awaiting confirmation (Child, 1992, 2004).

**New records:** This finding represents a new record for the Mediterranean Sea, and new country records for both Malta (#23: Figs. S11A and S11B) and Italy (#17, #18).

**Notes:** Recent molecular studies suggest that the Atlantic and Pacific populations may belong to different entities within a complex of species (Sabroux et al., 2017). Therefore, it is referred to here as *Achelia sawayai sensu lato*. Since the origin of the Mediterranean material is unknown, further molecular studies are necessary to understand the invasion route taken by this pycnogonid. The local reproductive success of this species exhibiting paternal care was demonstrated by the finding of two ovigerous male specimens.

## Discussion

This wide-scale study spanning the Mediterranean Sea provides a massive update of new NIS records, and in many cases their regional or local expansions, providing a warning for subsequent spreading. The 51 new country records presented in this study clearly indicate how inadequate our knowledge on Mediterranean marine NIS still is. There was a prevalence of new findings for bryozoans and crustaceans in almost all countries (Fig. 2). This is because these are both poorly studied taxa in the Mediterranean owing to a lack of taxonomic expertise/focused studies. Additionally, typical rapid assessment surveys or citizen science initiatives searching for NIS usually target larger and eye-catching taxa (Zenetos et al., 2013; Mannino et al., 2017), so these minuscule or less charismatic components of fouling biota may have gone previously overlooked or not have had the applicable expertise available.

It is not uncommon for marine NIS to go overlooked for long periods of time (Carlton, 2009), as in the case of *Paraleucilla magna* in the Mediterranean (Longo, Mastrototaro & Corriero, 2007). Actually, for most “first country records” documented in this study, the year of first introduction may have been much earlier than the first year of discovery presented here, as these NIS may have gone unnoticed due to a lack of taxonomic expertise or lack of focused study. Some probable examples of this include: *Paranthura japonica*, *Watersipora arcuata* and *Celleporaria brunnea*, whose current widespread Mediterranean distributions indicate they have likely been hitching rides around the basin for quite some time. Another example of this is the sea spider *Achelia sawayai sensu lato*, first reported here for the Mediterranean basin, and specimens were already found in three marinas: two in Sicily and one in Malta. An exception to this is our finding of *Microcosmus exasperatus* in Karpaz Gate Marina, Cyprus, as this species was specifically sought two years prior to our sampling of the same marina, but was not found then (Gewing et al., 2016). Also, *Celleporaria brunnea* was not found to be present in Grand-Motte, France in 2014 (Marchini, Ferrario & Minchin, 2015), but was present there when we sampled in 2016.

In addition to the records presented here, *Percnon gibbesi* (H. Milne Edwards, 1853) was sighted in Port Vauban, France, presenting a new country record for this species of western Atlantic origin, and already known from several other Mediterranean countries as a very successful invader (Katsanevakis et al., 2010, 2011). The non-indigenous status of *Percnon gibbesi* in the Mediterranean Sea is uncertain, because its long-lived planktonic larvae could have entered the Gibraltar Strait facilitated by natural means, i.e. the Atlantic Current, rather than human vectors, such as ballast water (Mannino et al., 2017 and references therein). Due to its questionable status regarding its mode of introduction, we have cautiously separated this species from the other NIS records. However, it is noteworthy that *Percnon gibbesi* was sighted feasting on a fouling community on a boat-hull in Greece (#25), suggesting hull-fouling as another possible vector for its ongoing spread.

This study focusing exclusively on marina habitats indicates that recreational boating represents the most plausible vector of introductions for the NIS we found, aside from the few marinas situated in very close proximity to either aquaculture facilities or shipping ports, as in Port Camargue, France and Heraklion, Greece. Hence, most of these new records suggest the pivotal role of recreational boating in facilitating both first introduction events to a given country and secondary spread.

Furthermore, some species reported here are likely polyvectic, but it is clear that recreational boating plays a determinant role in accelerating/facilitating the spread of many species, especially those having only a very short and lecithotrophic larval stage. The presence of such species lacking the ability for natural long-distance dispersal found on boat-hulls and in marinas can confirm that the hull-fouling vector is instrumental in expediting primary introductions as well as facilitating secondary transfer for many ascidians, bryozoans and peracarids such as *Ampithoe bizseli*, *Bemlos leptocheirus*, *Celleporaria brunnea*, *Clavelina oblonga*, *Paraleucilla magna*, *Paracerceis sculpta*, *Paranthura japonica, Phallusia nigra, Styela plicata* and *Tricelleria inopinata*. The ongoing nature of the invasion process is further demonstrated by the observation of the same set of NIS on boat-hulls and in the same marinas, clearly showing the exchange of organisms from marina to mobile habitats and *vice versa*.

The species which are not yet present in a country, but found only on boats obviously cannot formally be recorded as new country records, unless we are certain that the boat has not left that country since its last hull-painting/cleaning, e.g., as in our finding of *Paradella dianae* on boat-hulls in Greece. Some other interesting cases of NIS found on boats but not yet in the country are (see Table 3 for details) the barnacle *Amphibalanus improvisus* and the bryozoan *Watersipora arcuata* both found on hulls in France, yet the boats which they were found on had only travelled to the Balearic Islands, alluding to the assumption that those NIS are likely present in the Balearic Islands. Also noteworthy is the finding of the oyster *Saccostrea glomerata* from a boat-hull in France, representing a new record for the Western Mediterranean, and of the bryozoan *Parasmittina egyptica* from a boat-hull in Italy, representing the first Central Mediterranean record for this species. Overall, the 20 records presented in this study of NIS attached to boats but not yet recorded in the respective marina illustrates the potential of the biofouling vector in seeding a new area with propagules.

In synthesis, a pool of NIS is circulating among Mediterranean marinas, linked by a dense network of boat voyages ensuring their dissemination by a steady multiplication of the number of occasions. It is also of interest to point out that nearly all marinas have a rule prohibiting the in-water cleaning of vessels, but this rule is genuinely not enforced, and in-water cleaning was commonly witnessed within marinas during this study, likely facilitating the ‘stepping stone’ invasion process by dislodging and exacerbating the resettlement of NIS propagules.

Recently, Ferrario et al. (2017) showed that marinas can host as many NIS as larger commercial harbors. This massive contribution of new NIS records confirms their result and reveals that Mediterranean marinas so far have been inadequately explored for NIS, despite the Mediterranean Sea being both the global hotspot for boating traffic, and for level of NIS invasions. We strongly recommend that major attention should soon be dedicated to recreational marinas as hotspots of introduction, and to pleasure boats as a vector of introduction and spreading. Management actions to combat NIS in the Mediterranean Sea need to also incorporate the recreational boating vector.

## Supplemental Information

10.7717/peerj.3954/supp-1 Supplemental Information 1 Supplementary Data: Key Taxonomic Characters used for identification and Table with the number of visiting vessels per annum per marina.

10.7717/peerj.3954/supp-2 Supplemental Information 2 Supplementary Data: Specimen Photos.

## Definitions

Non-indigenous species: An organism introduced outside its natural past or present distribution range by human agency, either directly or indirectly (European Environment Agency, 2012).
Polyvectic species: May have been introduced by a certain combination of vectors and pathways (Carlton & Ruiz, 2005).
Pseudoindigenous species: A species described as new to an area where it was in fact introduced by human action (Carlton, 2009).

## Additional Information and Declarations

### Field Study Permissions

The following information was supplied relating to field study approvals (i.e., approving body and any reference numbers): The following people (including their position) authorised the field sampling permissions for this study: ***Spain*** Marina de Alicante: Seaman chief—Marìn One Ocean Port Vell Barcelona: Communications Officer—Regina Sorreno ***France*** Cap d’Agde: Marina Director—Pierre Weiss La Grand-Motte: Marina Director—Eric Pallier Port Camargue: Marina Director—Michele Cavalais Saint-Tropez: Marina Director—Jean-Francois Tourret Marines du Cogolin: Marina Director—Claude Robert Saint-Maxime: Marina Manager—Mr. Ellul Cannes Le Vieux Port: Marina Director—Francois Gillet Antibes Port Vauban: Marina manager—Emilie Pluenet Villefranche-sur-Mer: Chef d’Exploitation—Sarah Castanie ***Italy*** Porto Turistico di Roma/Lido di Ostia: Marina Manager—Daniele Cossu Ischia Marina di Sant’Angelo: Giulio Lauro; Porto d’Ischia: Mr. Di Maio Sorrento Marina Piccola: Italian Coast Guard/Sorrento Location Marina Villa Igiea Palermo: Marina Owner—Gioacchino Guccione Porto La Cala Palermo: Marina Manager—Nicola Rossa Porto Grande Siracusa: The Italian Coast Guard/Siracusa granted verbal permission. Marina di Marzamemi: Marina Director—Salvatore Gurrieri Marina di Ragusa: Marina Manager—Enza Di Ramondo Marina di Cale del Sole (Licata): Marina Manager—Maria Sitibondo Porto dell’Etna/Marina di Riposto: Marina Manager—Emiliano Indelicato ***Malta*** Msida Yacht Marina: Marina Director—George Mercieca Grand Harbour Marina Valletta: Marina Director—Gordon Vassolo ***Greece*** Old Venetian Harbour (Heraklion): Marina Director—Dr. Bras Ioannis Agios Nikolaos Marina: Marina Manager—Mikhalis Farsaris and Marina Director—Garefallakis Michalis. Rhodes Mandraki Port: Chief of the Port Police—Mr Moustakopoulos Ioannis ***Turkey*** Istanbul Atakoy Marina: Marina Manager—Asli Ebru Erkoc Istanbul Setur Kalamiş Marina: Marina Manager—Kerem Cesmebasi Milta Bodrum Marina: Marina Communications Director—Ayşe Mine Aykutluğ Setur Marmaris Netsel Marina: Marina Director—Erkan Ozatag & Marina manager—Onur Kunduz Datça Marina: Marina Manager—Ali Gök Ece Fethiye Marina: Marina communications officer—Mrs. Yelena Setur Finike Marina: Marina Manager—Zia Dal ***Cyprus*** Karpaz Gate Marina: Marina Director—Deniz Akaltan Famagusta Port: The Turkish Coast Guard granted verbal permission with Erol Adelier’s and Burak Çiçek’s assistance.

### Data Availability

The following information was supplied regarding data availability: The data that this output produced is all detailed in Tables 2, 3 and 5, with details of each species in the Results.
