# Correction: A massive update of non-indigenous species records in Mediterranean marinas

**DOI:** 10.7717/peerj.3954/correction-1

**Published:** 2017-11-14

**Authors:** Aylin Ulman, Jasmine Ferrario, Anna Occhpinti-Ambrogi, Christos Arvanitidis, Ada Bandi, Marco Bertolino, Cesare Bogi, Giorgos Chatzigeorgiou, Burak Ali Çiçek, Alan Deidun, Alfonso Ramos-Esplá, Cengiz Koçak, Maurizio Lorenti, Gemma Martinez-Laiz, Guenda Merlo, Elisa Princisgh, Giovanni Scribano, Agnese Marchini

**Affiliations:** 1 Department of Earth and Environmental Sciences, University of Pavia, Pavia, Italy; 2 Laboratoire d’Ecogéochimie des Environnements Benthiques, Université Pierre et Marie-Curie, Banyuls-sur-Mer, France; 3 Institute of Marine Biology, Biotechnology and Aquaculture, Hellenic Center of Marine Research, Heraklion, Crete, Greece; 4 Dipartimento di Scienze della Terra dell’Ambiente e della Vita (DISTAV), Università degli Studi di Genova, Genova, Italy; 5 Gruppo Malacologico Livornese, Livrono, Italy; 6 Department of Biological Sciences, Eastern Mediterranean University, Famagusta, Cyprus; 7 Department of Geosciences, University of Malta, Msida, Malta; 8 Marine Research Centre (CIMAR), University of Alicante, Alicante, Spain; 9 Department of Hydrobiology, Faculty of Fisheries, Ege University, Izmir, Turkey; 10 Center of Villa Dohrn-Benthic Ecology, Stazione Zoologica Anton Dohrn, Ischia, Italy; 11 Department of Zoology, University of Seville, Seville, Spain

Corrigendum:

After publication, a reader suggested that the text makes unnecessary reference to the political division of Cyprus. As the text is concerned with geography, rather than political borders, we have corrected this. The following changes have been made: 

Burak Ali Çiçek’s affiliation is now listed as Department of Biological Sciences, Eastern Mediterranean University, Famagusta, Cyprus. 

In the second paragraph of the Materials and Methods, “including the Turkish part of the island of Cyprus thereafter, only “Cyprus” for simplicity” has been replaced with “and Cyprus.”

The NIS record for Septifer cumingii Récluz, 1848 has been deleted as it was already reported in Crete in 2011. 

In the notes for Saccostrea cucullata, we referred to this species incorrectly as Septifer culcullata. Septifer cucullata has been replaced with Saccostrea cucullata in the NIS records for Saccostrea cf. cuculatta and Saccrostrea glomerata.

