# Supplementary material for: A massive update of non-indigenous species records in Mediterranean marinas
Source: PeerJ. 2017 Oct 24;5:e3954. doi: 10.7717/peerj.3954 (PMC5659216; doi:10.7717/peerj.3954)
Supplement: Supplemental Information 1 [file peerj-05-3954-s001.docx]

**Key Taxonomic Characters for New Alien Species Records**

**Ascidiacea**

## Family Ascidiidae

## ***Diplosoma listerianum*** (Milne-Edwards 1841)

**Key taxonomic characters**: Colonial ascidian with immersed zooids. Forms thin, flat, jelly-like transparent or milky-white sheets less than 2 mm think. Common tunic without spicules, it may contain grey or brown pigmented flecks. Zooids with fixator's appendix. Esophagus and intestine crossed. Gonads to left of gut, ovary with more than two oocytes.

## ***Phallusia nigra*** Savigny, 1816

**Key taxonomic characters**: Solitary ascidian with a very smooth, jet or ink-black tunic (can also be dark brown) devoid of epibionts, however juvenile specimens appear greyish. Long, curved oral siphon, bringing it near short atrial siphon, up to 10 cm in length. After alcohol preservation, the tunic turns a dark blue hue. Removal of tunic exposes a dense network of longitudinal and tranverse muscular bands on right side.

## Family: Clavelinidae

## ***Clavelina oblonga*** Herdman, 1880

**Key taxonomic characters**: Colonial ascidian, joined at base to others by short stolons. Soft tunic, mostly transparent with white speckled dots. On thick basal tunic, numerous fine stolons ending in white pigmented budding chambers. Zooids 25 mm in length, with some white pigment in branchial sac and stomach, but can also be blue or pink. 20 simple tentacles of various orders, vertical oval neural gland aperture and about 20 rows of stigmata in the branchial sac (with 50-60 stigmata per half row). Digestive system contains a descending esophagus, a subterminal squared stomach with marked ridges, followed by a mid-intestine and an ascending rectum. The gonads left of intestinal loop, and stomach dorsally containing numerous ovoid and small male follicles, with a mass of oocytes in middle of testes (Ordóñez et al. 2016; Rocha et al. 2012).

## Family: Pyuridae

***Microcosmus exasperatus*** Heller 1878 and ***Microcosmus. squamiger*** Michaelsen, 1927

**Key taxonomic characters**: Solitary ascidian with hard tunic. Globular and irregular shape with prominent siphons. Brown in colour sometimes with small epibionts. Up to 4 cm in length. Found in small clusters, not dense aggregations. Two species impossible to distinguish without dissection (See remarks below).

**Remarks:** *Microcosmus exasperatus* is differentiated taxonomically from *M. squamiger* by the shape of the siphonal spines from the inhalant siphon (Kott 1985), which are much longer and pointy (and curved like a shark fin, Kott 1985) in *M. exasperatus* and shorter and rounded at the top (‘roma’ shaped) in *M. squamiger* (Ramos-Espla et al. 2013; Turón X et al. 2007).

## Family: Styelidae

***Styela plicata*** (Lesueur, 1823)

## **Key taxonomic characters**: Solitary, unstalked ascidian. Externally, can be whitish to tan or greyish in color, often containing epibionts; tunic quite lumpy, although juveniles are less lumpy in appearance but have small pleats. Internally, from 4 to 8 gonads on left and 2 on right (Ramos-Esplá 1988).

## ***Symplegma brakenhielmi*** (Michaelsen ~~n~~, 1904)

**Key taxonomic characters**: Encrusting colonial ascidian, zooids joined by common base. Thin transparent tunic with many vascular processes. Branchial sac with 4 longitudinal lines. Long and curved caecum connected to main intestinal tract with two bands of tissues; 4-5 longitudinal stomach plications. Testes with lobes (Kott 1985; Monniot & Monniot 2001).

**Bryozoa**

## Family: Candidae

***Tricellaria inopinata*** d'Hondt & Occhipinti Ambrogi, 1985

## **Key taxonomic characters**: Dichotomously branched whitish arborescent colonies. Well-marked joints at the bases of the rami. Autozooids disposed in alternating series, bearing avicularia with jaw-like mandible. Autozooids with spines, generally three in the outer and two in the inner side. Scuta usually small, variable in shape and size within the colony.

Family: Hippopodinidae

## ***Hippopodina* aff. *feegeensis*** Tilbrook, 2006 (Red Sea material)

**Key taxonomic characters**: Unilaminar encrusting colonies, autozooids rectangular in shape with the frontal wall perforated by numerous small pores. Primary orifice bell-shaped, with proximal border shallowly concave (which is generally straight in *H. feegeensis*); orifice with medium-sized lateral indentation, poster 90% width of anter (80% in *H. feegeensis*). Single or paired medium-sized adventitious avicularia, elongated-triangular in shape and medially directed.

## Family: Lepraliellidae

## ***Celleporaria brunnea*** (Hincks, 1884)

**Key taxonomic characters**: Unilaminar or multilaminar encrusting irregular colonies. Opercula, sclerites of avicularia mandibles, base of spines and the lophophore tentacles dark-brown in colour. Orifice proximal border with midline notch and small horizontal condyles. Peristome usually with black joined spines. Suboral and large interzooidal avicularia present.

## ***Celleporaria vermiformis*** (Waters, 1909)

**Key taxonomic characters**: Multilaminar encrusting colonies black in colour. Autozooids with large marginal pseudopores, subcircular primary orifice with condyles. Orificial spines lacking. Both small adventitious and gigantic vicarious avicularia present.

**Remarks:** *Celleporaria vermiformis* specimens analyzed in this study differ from specimen described in Harmelin (2014) in having an orifice with condyles, less concave proximal edge, shorter suboral umbo, lower and narrower ovicell and presence of gigantic vicarious avicularia. Specimens described here more similar to those of *C. vermiformis* from Safaga N Bay (Red Sea) by Ostrovsky et al. (2011); while the Lebanese specimen described by Harmelin (2014), under the name *C. vermiformis*, seems to be more similar to *Celleporaria melanodermorpha* Liu, 2001 (J.-G. Harmelin personal communication).

Family: Smittinidae

***Parasmittina egyptiaca*** (Waters, 1909)

**Key taxonomic characters**: Unilaminar encrusting colonies, generally small. Autozooids generally arranged in regular rows, with large marginal pores. Primary orifice rounded with 2-3 spines. Lyrula broad, with distal edge straight and the sides at 45°. Two condyles digitate with denticulate tips. Peristome interrupted distally. Polymorphic adventitious avicularia variable in number. Less frequently a single gigantic spatulate avicularium is present, facing the lateral side of the autozooids with triangular flaps.

## Family: Vesiculariidae

## ***Amathia verticillata*** (delle Chiaje, 1822)

**Key taxonomic characters**: Stoloniferous fouling bryozoan with bushy or more elongated colonies; irregularly-branching arrangement can exceed one meter in length. Colony translucent, zooids oval in shape and, in young branches, arranged on two regular parallel rows, while irregularly arranged on the basal branches.

Family: Waterisporidae

***Watersipora arcuata*** Banta, 1969

**Key taxonomic characters**: Unilaminar or multilaminar encrusting colonies, transparent to reddish-brown/black in colour. Operculum and orifice semicircular, with the orifice proximal margin curved inward. Cardelles (projections of the border of the orifice) present at about one-third (or more proximally) of the orifice length. Pair of transparent opercular lucidae present and frontal wall perforated by pseudopores. *Watersipora* genus characterized by the absence of spines, avicularia and ovicells.

**Crustacea**

Cirripedia

## Family: Balanidae

## ***Amphibalanus improvisus*** (Darwin 1854)

**Key taxonomic characters**: Maximum 17 mm diameter. Plates white and smooth with slightly toothed orifice. Plates have white longitudinal radii narrowing as they reach the top. Scutum with well-developed adductor ridge on interior face (Zullo 1979), while tergum has blunt apex and spur long and narrow. Spur length about 1/3 length of basal margin, width about 1/5 of basal margin (Henry & McLaughlin 1975).

***Balanus trigonus*** Darwin 1854

**Key taxonomic characters**: Maximum 25 mm diameter, triangular aperture, six external plates, purplish to pink with white striations. Distinguishable by scutum, with 1 to 6 longitudinal rows of pits, while tergum is broad, smooth and flat.

- 1. Decapoda
  2. Family: Portunidae
  3. ***Charybdis (Gonioinfradens) paucidentatus*** (A. Milne-Edwards, 1861)
  4. **Key taxonomic characters**: Maximum length of 52.5 mm with a hexagonal, smooth carapace. Six teeth on front, with median and submedian teeth truncate, lateral teeth triangulate and rounded at tip, separated by deeper groove from previous ones. With four large anterolateral teeth, first is more rounded and last spiniform. Two accessory denticles at base of external border of first and second teeth, second very small. Chelipeds have three strong spines on anterior border, carpus with strong interior spine and 3 smaller spines on outer face, chela has two large spines on superior surface and two other marginal spines near movable finger (Corsini-Foka et al. 2010).
  5. **Remarks:** The presence of only four large anterolateral teeth allows *Gonioinfradens* to be easily distinguished from all the other subgenera retained in *Charybdis* (Apel & Spiridinov 1998).

## Family: Portunidae

## ***Dyspanopeus sayi*** (Smith 1969)

**Key taxonomic characters**: Maximum carapace width of 30 mm. Live specimens greenish to brown with reddish dots on dorsal side, creamy ventral side. Oval, arcuate carapace, small median notch on front, minutely granular. Five teeth on each anterolateral margin, first two coalescent and near ocular lobe margin, last three prominent but variable in shape. Walking pereiopods have long and slender dactylus; pereiopods 2 to 5 are shorter than pereiopod 1. First male pleopod has a low mesial lobe which is broadly rounded, differing it from *D. taxana* which has an elongate and narrow mesial lobe. Chelipeds unequal in size in male only, chelipeds with small tubercles. Fingers of chelae variable in colour, from ivory to dark brown to black.

Peracarida - Amphipoda

Family: Ampithoidae

***Ampithoe bizseli*** Özaydinli & Coleman, 2012

## **Key taxonomic characters:** Gnathopods (Gn) 1 and 2 with rounded lobes on basis anterodistal corner and ischium anterior margin (small in females, large in males). Particularly, male Gn2 lobe very large and reaches beyond ischium. Male Gn2 propodus bearing a prominent anterodistal lobe, with long setae; palm excavate, defined by posterodistal tooth.

## Family: Aoridae

## ***Aoroides longimerus*** Ren & Zheng 1996

**Key taxonomic characters (males only)**: Gnathopod (Gn)1 long, merochelate, densely setose, much larger than Gn2. Coxa bearing a few plumose setae and spine. Basis elongated, bearing dense plumose setae along the anterior margin and some setae in lateral margin. Maerus bearing long plumose setae, prolonged into a long distal tooth extending way beyond the carpus; tip abruptly narrowed. Carpus large, ventral and medial surface with long plumose setae. Propodus bearing simple setae at anterodistal corner and along posterior margin. Dactylus robust, long, curved, bearing several simple setae along posterior margin. Gn2, basis with long simple setae and a plumose seta in anterior margin, a few simple setae in posterior margin. Carpus and propodus with simple setae along posterior margin. Propodus with transverse palm, posterior margin with a spine. Uropod 3 biramous.

***Bemlos leptocheirus*** (Walker, 1909)

**Key taxonomic characters:** Antenna 1 with 5-articulated accessory flagellum. Gnathopod (Gn) 1 bigger than Gn2; Gn1 carpus shorter than half the length of propodus, propodus subchelate, with long setae and (in males) a triangular tooth at posterodistal margin. Gn2 merus, carpus and propodus bringing long plumose setae; carpus and propodus elongated and slender. Third uropod uniramous

Family: Ischyroceridae

## ***Erichthonius* cf. *pugnax*** (Dana 1852)

**Key taxonomic characters (males only)**: Gnathopod (Gn) 2 very large and carpochelated; coxa with stridulating ridges, carpus with two posterodistal teeth, the outer being longer (while hyper-adult males exhibit a single prominent tooth); propodus shorter than carpus; dactylus slender, with apical tuft of long setae. Pereopods 3-4 basis flask-shaped; pereopod 5 basis with a distinctive lobe on the posterodistal margin. Posterior margin of epimeral plate 3 minutely serrated.

Family: Stenothoidae

## ***Stenothoe georgiana*** Bynum & Fox 1977

**Key taxonomic characters (males only)**: Gnathopod (Gn) 1 carpus triangular, propodus with weakly convex palm, finely denticulate, bringing a few spines. Gn2 propodus palm defined by a characteristic spinose hump (weak in females, prominent in males); dactylus reaching palmar hump. Telson with two rows of longitudinal spines.

Peracarida - Isopoda

## Family: Anthuridae

## ***Mesanthura* sp.** Lorenti, Dappiano & Gambi, 2009

**Key taxonomic characters**: This new species is awaiting proper description. *Mesanthura* genus only one amongst anthuridean isopods, along with *Chelanthura*, exhibiting species-specific pigmented patterns (Poore, 2001). The species reported here present a characteristic pigmented dorsal pattern arranged in composed patches on head and pereonites, with interruptions of blank areas. Pleon contains five stripes, delimited by two semi-circles, a narrower anterior one and a wider distal one; patches of pigment also cover telson and uropods. Morphological features of diagnostic relevance include, the presence of 6-7 spines on the distal mandibular palp article, the palm of pereopod 1 with a step, the broadly notched uropod exopod.

**Remarks:** Our material is obviously conspecific with the species recorded by Lorenti et al. (2009) from two Italian harbors (Maurizio Lorenti, personal obs.) and compared to the Australian species *M. romulea* primarily based on similar identity of cephalic and pereional decoration.

## Family: Janiridae

## ***Ianiropsis serricaudis*** Gurjanova, 1936

**Key taxonomic characters**: Elongate segments 6 and 7 of antennal peduncle. Maxilliped palps elongated and visible in dorsal view. Pereopod 1 with two claws on dactylus, and three claws on peraeopod 7. Pleotelson lateral margin with three or four denticles along the posterior half.

Family: Sphaeromatidae

## ***Cymodoce* aff. *fuscina*** Schotte & Kensley 2005

**Key taxonomic characters (males only)**: Pereon weakly setose laterally and with tufts of dorsal setae, bearing dorsal tubercles on the three distal segments. Pleotelson densely setose and rugose, ending in a deeply notched tridentate apex. Proximal part of pleotelson with two prominent longitudinal ridges, flanked by bifid distal tubercoles. Uropod rami extending beyond the pleotelsonic medial lobe; endopod with two clearly visible dorsal tubercles. Appendix masculina straight.

***Paradella dianae*** (Menzies, 1962)

**Key taxonomic characters**: Males: segments 5-7, distal margin dorsally protruding (visible in lateral view). Pleotelson granulated, with two pairs of prominent tubercles, Pleotelson ending with a characteristic heart-shaped indentation. Uropods enlarged, finely crenulated and surrounded by short setae. Females lack the prominent distal ridges in segments 5-7 and the heart-shaped indentation of the pleotelson, reduced to a weak depression.

***Paracerceis sculpta*** (Holmes 1904)

**Key taxonomic characters (males only)**: Easily distinguished from other sphaeromatid isopods by shape of pleotelsonic region. Pleon large, granulated, bearing three tubercles in distal margin. Pleotelson large, also with three tubercles, granulated in anterior part and setose in distal margin. Pleotelsonic apex cleft, with six notches, middle ones deeper than lateral ones. Uropod endopods flattened and short; exopods markedly elongated, cylindrical, with acute apex.

## Family: Paranthuridae

## ***Paranthura japonica*** Richardson 1909

**Key taxonomic characters**: Elongated body, covered with scattered pigmentation. Cephalon with anterolateral lobes extending beyond rostrum; mouth appendages produced in an acute piercing/sucking apparatus. Pereon segment 5 slightly longer than 6 and 7. Pleon segments fused dorsally but not laterally.

## ***Sphaeroma walkeri*** Stebbing 1905

**Key taxonomic characters**: Pereon and pleotelson dorsally granulose, with rows of tubercles along posterior margins of pereonites, and four parallel rows of tubercles on pleotelson, directed longitudinally. Uropod endopod also with two or three prominent tubercles; exopod outer margin deeply serrated. Telson with denticulate posterior margin.

**Mollusca**

## Family: Chamidae

## ***Pseudochama* cf. *corbierei*** (Jonas 1846)

**Key taxonomic characters**: Shell extremely inequivalve, lower valve (rv) deeply concave and upper valve quite flat with large area to attach to substrate. Irregular outline, exterior structure often eroded, can be subcircular. Very similar to *Pseudochama gryphina* but *P. corbieri* has a distinctly thicker shell. In addition, the exterior sculpture consists of fine to slightly roughened concentric, close-set chords. The interior colouration especially in upper valve suffused with purple, and crenulated margins, which are key identification characters (Delsaerdt 1986). Lower point of shell’s initial coiling in lower valve reaches to about 42% of total shell length, which is only about 25% in *P. gryphna*.

**Remarks:** Lower point of the shell’s initial coiling in the lower valve reaches about 25% in the comparable *P. gryphina*, and exterior sculpture is more scale-like.

## Family: Mytilidae

## ***Arcuatula senhousia*** (Benson 1842)

**Key taxonomic characters**: Shell reaches a maximum 30 mm length, shell colour ranges from yellow-brown to dark-brown and is covered by a greenish periostracum. Has to 6 to 8 clearly visible ribs anterior to the umbone and is also accompanied by light-coloured radiating lines (Hoenselaar & Hoenselaar 1989). Ventral margin a little bit concave, widened posteriorly. Anterior margin crenulated.

Family: Mytilidae

***Septifer cumingii*** Récluz, 1848

**Key taxonomic characters**: Up to about 10 mm in length and 5 mm in width. Shell equivalve with subtriangular shape. Presence of subterminal umbones near anterior margin. Strong radial ribs, sometimes forked, crossed by finer concentric lines. Internal septum crossing umbonal cavity key identification feature. Hinge has 2-3 large teeth below umbo, and 4-6 large teeth behind ligament. Periostratum bristly all over surface, light-green in colour with pink or orange spots.

## Family: Ostreidae

## ***Dendostrea* *folium sensu lato*** (Linneaus 1758)

**Key taxonomic characters**: Highly variable morphology and colouration, often assuming nature of substrate. Foliate oyster, up to 60 mm in length, variable in colour, including brownish, whitish, reddish, pinkish. Thin, elongate oval; margin irregularly folded. Both valves concave having dichotomous radial ribs from umbo, top of ribs rounded. The submedian ridge not always present and number of plications is highly variable. Both valves with many fine and imbricate growth squamae, sometimes eroded dorsally. Adductor muscle scar kidney shaped, few chomata. Commissural shelf narrow. Umbonal cavity shallow.

## ***Saccostrea* cf. *cucullata*** (Born 1778)

**Key taxonomic characters**: Up to 60 mm length in Mediterranean. Shell inequivalve with lower valve (left valve, lv) larger, and can be deeply cupped, while right valve almost flat with plicated margin fitting margins of lower valve. Interior shell margin of right valve (rv) with prominent denticles fitting pits of lv margin. Sculpture highly variable from smooth, to strong radial ribs, and even spines. Shell also variable in outline, from nearly circular to oblong or roughly oval. Hinge untoothed. No sculpture at umbo.

Remarks: *Saccostrea* sp. are crenulated for entire perimeter whereas *Ostrea* sp. only have anterior margin near hinge crenulated. Indo-Pacific specimens reach larger dimensions (130 mm).

## ***Saccostrea glomerata*** (Gould 1850)

**Key taxonomic characters**: From 70-100 mm in length. Lower valve deep and cupped with weakly crenulated margin, flattened upper valve and folds towards lip to fit crenulations from lower valve. Presence of small denticles along edge near hinge which are closer together on lower valve compared to upper valve. Upper valve often has nodular ribs separated by large grooves.

**Polychaeta**

Family: Serpulidae

## ***Hydroides brachyacantha*** ***sensu lato*** Rioja 1941

**Key taxonomic characters**: White calcaereous tubeworm. Opercular verticil possessing approximately 8 dark-brown strongly curved inward spines, dorsal hook longer and wider than others, spines covering the central disc, other spines similar to each other in size and shape (hooks near dorsal hook slightly larger than others). Spines have one short internal basal spinule (Çinar et al. 2006; Sun et al. 2016).

## ***Hydroides dirampha*** Mörch, 1863

**Key taxonomic characters**: White calcaereous tubeworm up to 36 mm in length (16 mm on average). Opercular verticil possessing 11 to 15 spines, similar in size and shape, with a distinct T or arrowhead flattened shape at the tips, and with one basal internal spinule. Without central tooth (Bastida-Zavala & ten Hove 2003; Sun et al. 2015).

## ***Hydroides elegans*** (Haswell, 1883)

**Key taxonomic characters**: White calcaereous tubeworm, sub-trapezoidal in cross-section, maximum tube length of 80 mm, with two longitudinal ridges; maximum body length of 20 mm. Opercular verticil with short central tooth possessing 14-17 radiating spines, each having 2-4 lateral processes and a medial row with 1-4 short internal spinules. Collar chaetae: bayonet, with 2-4 short teeth and a rasp behind them (Bastida-Zavala & ten Hove 2003).

***Hydroides homoceros*** Pixell 1913

**Key taxonomic characters**: White calcaereous tube with three longitudinal ridges, less than 10 mm in body length. Opercular verticil chaeta possessing 6 spines, bayonet shaped with twinned spines in the middle of the chaetal length. Funnel with 18 radii, each with a pair of lateral spines pointed downwards, tips of radii T-shaped; Verticil with 6 spines of similar length, curved inwards with twinned lateral spinules at mid-length (Çinar 2006).

***Spirobranchus tetraceros*** ***sensu lato*** (Schmarda 1961)

**Key taxonomic characters**: Calcaereous tubeworm, pale purple in colour with three high longitudinal ridges and many transversal ridges. Broad peduncle with lateral wings crenulated on their inner distal margins; operculum flat with three long branching antler-like spines. Collar chaetae including 5 bayonet, 10 limbate, striate chaetae covered with minute denticles (Çinar 2006).

Porifera

Family: Amphoriscidae

***Paraleucilla magna*** Klautau, Monteiro & Borojevic, 2004

**Key taxonomic characters**: Calcareous sponge varying in shape from tubular to massive or irregular. Oscula located at ends of the tubes. Compressible consistency but sponge is friable, with smooth surface. Live specimens creamy-white coloured, not changing after alcohol preservation. Triactine and tetractine spicules.

Pycnogonida

Family: Ammotheidae

***Achelia sawayai* *sensu lato*** Marcus, 1940

**Key taxonomic characters**: Trunk segments fused, outline circular; lateral processes touching or slightly separated, without major tubercles. Ocular tubercle height similar to width; large eyes, distinctly pigmented. Chelifor scapes one segmented; chelae vestigial, globular. 8 segmented palps; 4 terminal short segments, very setose; 2^nd^ and 4^th^ segments longest. 10 segmented ovigers, with weak strigilis bearing denticulate spines; 4^th^ and 5^th^ segments longest. Legs moderately slender; coxae 1 with 3 and coxae 2 with 2 laterodistal tubercules, terminating in one short seta; cement gland tube cone shaped at dorso-distal of femur, ending in very short tube-shaped duct; propodus large, slightly curved, without heel.

**Notes:** This species and *Achelia besnardi* Sawaya, 1951, known from the western Atlantic, are very similar except from the lateral processes of *A. besnardi* have conspicuous tubercles and the leg segments are relatively longer and more slender. Both the male and female of *A. besnardi* have fairly long dorso-distal tubercles on the femorae, almost as long as the femur diameter. The trunk of *A. besnardi* is not quite circular in outline, and the lateral processes do not always touch (Child, 1992). *Achelia sawayai* is also similar to *A. gracilis* Verrill, 1900, which is known from western Atlantic, and can be confused with this species. The dorso-distal tubercles on the lateral processes in the males of *A. gracilis* are stronger and coxa 1 of legs 1–2 show 4 tubercles as opposed to 3 with *A. sawayai*. A clear identification character in both sexes is the number and shape of palp articles of both species (7 in *A. gracilis*: 8 in *A. sawayai*) (Müller & Krapp, 2009). This study shows that this sea spider is likely establishing itself around Malta and Sicily, as one male specimen was ovigerous.

| **Supplementary Table 1.** Number of visiting vessels per marina per annum (for either 2014, 2015 or 2016).  2015 or 2016) | | | |
| --- | --- | --- | --- |
| 1. Alicante, Spain | N/A | 18. Siracusa, Italy | N/A |
| 2. Barcelona, Spain | 2122 | 19. Marzememi, Italy | N/A |
| 3. Cap d'Agde, France | 1000 | 20. Ragusa, Italy | N/A |
| 4. La Grand-Motte, France | N/A | 21. Licata, Italy | 500 |
| 5. Port Camargue, France | N/A | 22. Msida, Malta | 350 |
| 6. Saint-Tropez, France | 700 | 23. Grand Harbour, Malta | 459 |
| 7. Cogolin, France | N/A | 24. Heraklion, Greece | N/A |
| 8. Saint-Maxime, France | 1221 | 25. Agios Nikolaos, Greece | N/A |
| 9. Cannes, France | N/A | 26. Rhodes, Greece | N/A |
| 10. Antibes, France | 1200 | 27. Istanbul, Turkey | N/A |
| 11. Villefranche-sur-Mer | 2000 | 28. Bodrum, Turkey | N/A |
| 12. Rome, Italy | N/A | 29. Datça, Turkey | 450 |
| 13. Ischia, Italy | N/A | 30. Marmaris, Turkey | 2000 |
| 14. Sorrento, Italy | 1200 | 31. Fethiye, Turkey | 1500 |
| 15. Villa Igiea, Italy | 476 | 32. Finike, Turkey | 2500 |
| 16. La Cala, Italy | N/A | 33. Karpaz, Cyprus | 300 |
| 17. Riposto, Italy | 800 | 34. Famagusta, Cyprus | 50 |

**References**

Apel M, and Spiridinov VA. 1998. Taxonomy and zoogeography of the portunid crabs (Crustacea: Decapoda: Brachyura: Portunidae) of the Arabian Gulf and the adjacent waters. *Fauna of Arabia* 17:159-331.

Bastida-Zavala JR, and ten Hove HA. 2003. Revision of *Hydroides* Gunnerus, 1768 (Polychaeta: Serpulidae) from the eastern Pacific Region and Hawaii. *Beaufortia* 53:67-110.

Çinar M. 2006. Serpulid species (Polychaeta: Serpulidae) from the Levantine coast of Turkey (eastern Mediterranean), with special emphasis on alien species. *Aquatic Invasions* 1:223-240. 10.3391/ai.2006.1.4.6

Çinar ME, Bilecenoglu M, Ozturk a, and Can A. 2006. New record of alien species on the Levantine coast of Turkey. *Aquatic Invasions* 1:84-90.

Corsini-Foka M, Pancucci-Papadoulou MA, Kondilatos G, and Kalogirou S. 2010. *Gonioinfradens paucidentatus* (A. Milne Edwards, 1861) (Crustacea, Decapoda, Portunidae): a new alien crab in the Mediterranean Sea. *Mediterranean Marine Science* 11:331-340.

Delsaerdt A. 1986. Revision of the Chamidae of the Red Sea. *Gloria Maris* 25:73-125.

Harmelin JG. 2014. Alien bryozoans in the eastern Mediterranean Sea - new records from the coast of Lebanon. *Zootaxa* 3893:301-338.

Henry D, and McLaughlin P. 1975. The barnacles of the *Balanus amphitrite* complex (Cirripedia, Thoracica). *Zoologische Verhandelingen* 141:203.

Hoenselaar HJ, and Hoenselaar J. 1989. *Musculista senhousia* (Benson in Cantor, 1842) in the western Mediterranean (Bivalvia, Mytilidae). *Basteria* 53:73-76.

Kott P. 1985. The Australian Ascidiacea. Part 1. Phlebobranchia and Stolidobranchia. *Memoirs of the Queensland Museum* 23:440.

Lorenti M, Dappiano M, and Gambi MC. 2009. Ocurrence and ecology of *Mesanthura* (Crustacea: Isopoda: Anthuridea) in two Italian harbours. *Marine Biodiversity Records* 2:e48.

Monniot F, and Monniot C. 2001. Ascidians from the tropical western Pacific. *Zoosystema* 23:201-383.

Ordóñez V, Pascual M, Fernández-Tejedor M, and Turon X. 2016. When invasion biology meets taxonomy: *Clavelina oblonga* (Ascidiacea) is an old invader in the Mediterranean Sea. *Biological Invasions* 18:1203-1215. 10.1007/s10530-016-1062-0

Ostrovsky A, Cáceres-Chamizo JP, Vávra N, and Berning B. 2011. Bryozoa of the Red Sea: history and current state of research. *Annals of Bryozoology* 3:67-97.

Ramos-Espla A, Izquierdo A, and Çinar M. 2013. *Microcosmus exasperatus* (Ascidiacea: Pyuridae), current distribution in the Mediterranean Sea. *Marine Biodiversity Records* 6:e89.

Rocha RM, Kremer L, and Fehlauer-Ale K. 2012. Lack of COI variation for Clavelina oblonga (Tunicata, Ascidiacea) in Brazil: Evidence for its human-mediated transportation?  *Aquatic Invasions* 7:419-424.

Sun Y, Wong E, Hove H, Hutchings PA, Williamson JE, and Kupriyanova EK. 2015. Revision of the genus *Hydroides* (Annelida: Serpulidae) from Australia. *Zootaxa* 4009:99. 10.11646/zootaxa.4009.1.1

Sun Y, Wong E, Tovar-Hernández MA, Williamson J, and Kupriyanova E. 2016. Is *Hydroides brachyacantha* (Serpulidae: Annelida) a widespread species? *Invertebrate Systematics* 30:41-59.

Turón X, Nishikawa T, and Rius M. 2007. Spread of *Microcosmus squamiger* (Ascidiacea: Pyuridae) in the Mediterranean Sea and adjacent waters. *Journal of Experimental Marine Biology and Ecology* 342:185-188.

Zullo VA. 1979. Marine flora and fauna of the Northeastern United States. Arthropoda: Cirripedia, NOAA Technical Report NMFS Circular NOAA. p 1-29.
