# Supplementary material for: A massive update of non-indigenous species records in Mediterranean marinas
Source: PeerJ. 2017 Oct 24;5:e3954. doi: 10.7717/peerj.3954 (PMC5659216; doi:10.7717/peerj.3954)
Supplement: Supplemental Information 2 [file peerj-05-3954-s002.docx]

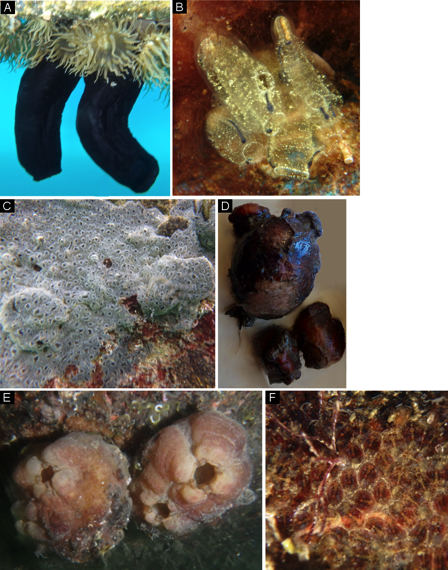


S.D. Fig. 1 (A-F). Ascidians: (A) *Phallusia nigra* in Marina #34; (B) *Clavelina oblonga* in Marina #34; (C) *Diplosoma listerianum* in Marina #23; (D) *Microcosmus squamiger* in Marina #20; (E) *Styela plicata* in Marina #14; (F) *Symplegma* cf. *brakenhielmi* in Marina #32. Photo credits: Aylin Ulman.


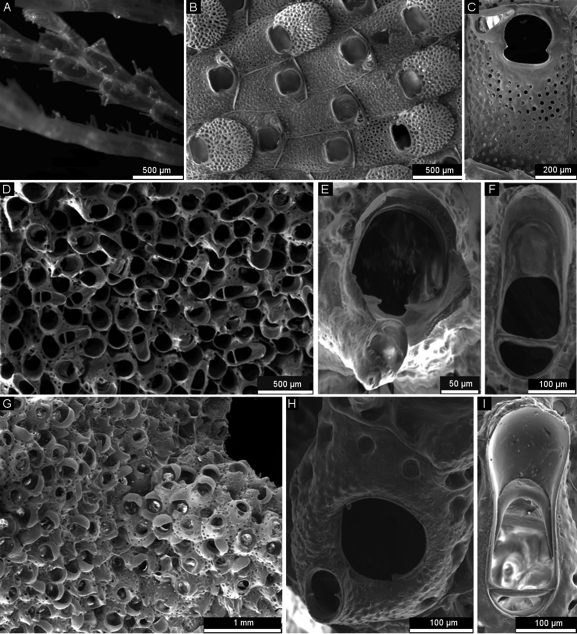


S.D. Fig. 2 (A-I). Bryozoans Part 1: (A) *Tricellaria inopinata* from Marina #5; (B-C) *Hippopodina* aff. *feegeensis*: (B) colony with ovicelled autozooids from Marina #26, (C) close-up of the autozooid with avicularia from Marina #32; (D-F) *Celleporaria brunnea* from Marina #5: (D) colony, (E) close-up of the orifice and the sub-oral adventitious avicularium, (F) close-up of the interzooidal avicularium; (G-I) *Celleporaria vermiformis* from Marina #33: (G) colony with ovicelled zooids; (H) close-up of the orifice and the sub-oral adventitious avicularium; (I) gigantic vicarious avicularium. Photo credits: Maria Pia Riccardi and Ilenia Tredici (CISRiC-Arvedi Laboratory) at the University of Pavia assisted Jasmine Ferrario with the use of the Scanning Electronic Microscope (SEM).


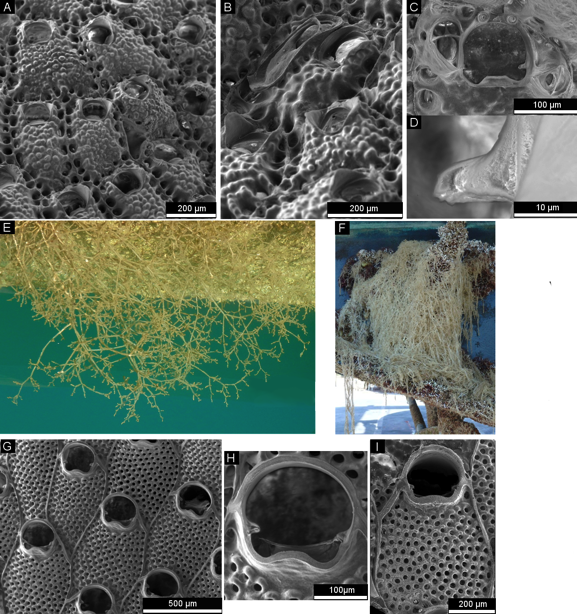


S. D. Fig. 3 (A-I). Bryozoans Part II: (A-B) *Parasmittina egyptiaca* on boat hull from Marina #25: (A) colony, (B) autozooid with a gigantic spatulate avicularium with triangular flaps; (C-D) *Parasmittina egyptiaca* from Marina #33: (C) close-up of the orifice with two small avicularia, (D) condyle; (E-F) *Amathia verticillata*: (E) colony in Marina #30, (F) colony on boat hull in Marina #12; (G-H) *Watersipora arcuata* from Marina #18: (G) colony, (H) close-up of the orifice; (I) *Waterispora arcuata* from Marina #22: autozooid. Photo credits: (A-C, G-I) Maria Pia Riccardi and Ilenia Tredici (CISRiC-Arvedi Laboratory) at the University of Pavia assisted Jasmine Ferrario with the use of the Scanning Electronic Microscope (SEM); (E, F) Aylin Ulman.


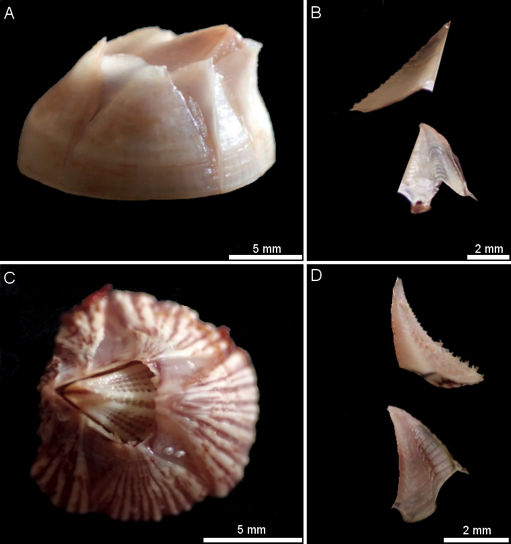


S.D. Fig. 4. (A-D). Cirripeds: (A-B) *Amphibalanus improvisus* on boat hull in Marina #5: (A) complete specimen, (B) scutum and tergum; (C-D) *Baluns trigonus* on boat hull in Marina #33: (C) complete specimen, (D) scutum and tergum. Photo credits: Aylin Ulman.


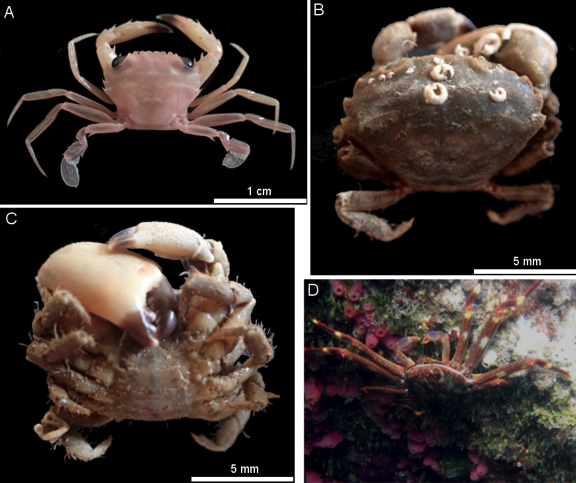


S.D. Fig. 5 (A-D). Decapods: (A) *Charybdis (Gonioinfradens) paucidentatus* in Marina #34; (B-C) *Dyspanopeus sayi* dorsal and ventral view in Marina #24; (D) *Percnon gibbesi* in Marina #24. Photo credits: Aylin Ulman.


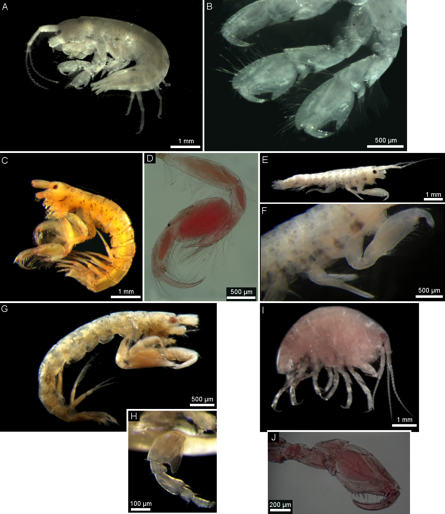


S. D. Fig. 6 (A-J). Amphipods: (A-B) *Amphithoe bizseli* in Marina #34: (A) male specimen, (B) right and left gnathopod 2; (C-D) *Aoroides longimerus* in Marina #5: (C) male specimen, (D) merochelate gnathopod 1; (E-F) *Bemlos leptocheirus* in Marina #24: (E) male specimen, (F) gnathopods 1 and 2; (G-H) *Ericthonius* cf. *pugnax* in Marina #5: (G) male specimen, (H) pereopod 5; (I-J) *Stenothoe georgiana* in Marina #14: (I) male specimen, (J) gnathopod 2 with conspicuous lobe on the propodus palm. Photo credits: (A-B) Agnese Marchini; (C-J): Gemma Martinez-Laiz.


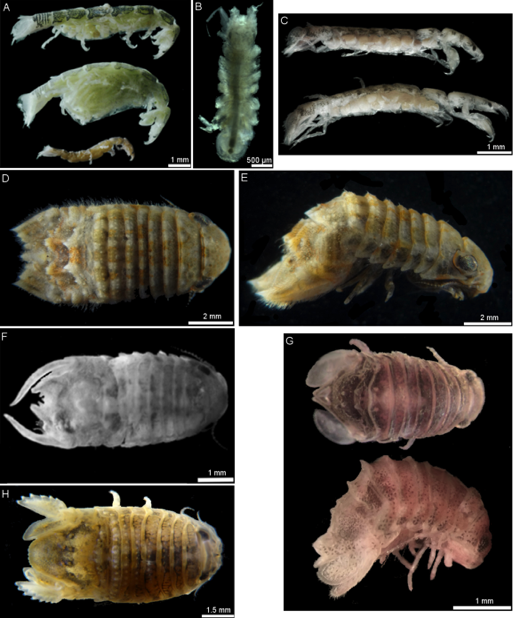


S. D. Fig. 7 (A-H). Isopods: (A) *Mesanthura* sp. in (from top to bottom) Marinas #1, #16 and #22: female specimens; (B) *Ianiropsis serricaudis* in Marina #5; (C) *Paranthura japonica* specimens in (from top to bottom) Marinas #1 and #21: female specimens; (D-E) *Cymodoce* aff. *fuscina* in Marina #24: (D) frontal and (E) lateral view of a male specimen, (F) *Paracerceis sculpta* in Marina #22: male specimen; (G) *Paradella dianae* in Marina #15: male specimen; (H) *Sphaeroma walkeri* in Marina #24: male specimen. Photo credits: (A, B, C); Agnese Marchini; (D, E, F, H): Gemma Martinez-Laiz; (G) Giovanni Scribano.


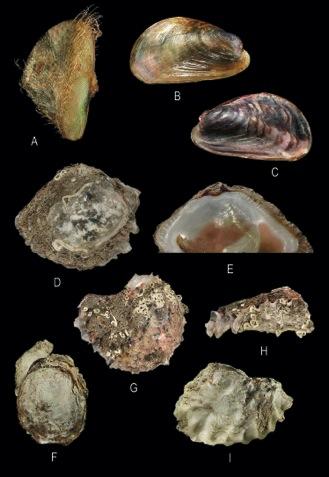


S.D. Fig. 8 (A-I). Molluscs: (A) *Septifer cumingii* in Marina #25, L= 8,5 mm; (B-C) *Arcuatula senhousia* in Marina #2, L=19 mm; (D-E) *Saccostrea glomerata* in Marina #10, L= 40 mm; (F) *Pseudochama* cf. *corbierei* in Marina #20, L= 21 mm; (G-H) *Saccostrea* cf. *cucullata* in Marina #24, L= 25 mm; (I) *Dendostrea folium sensu lato* in Marina #33, L= 25mm. Photo credit: Cesare Bogi.


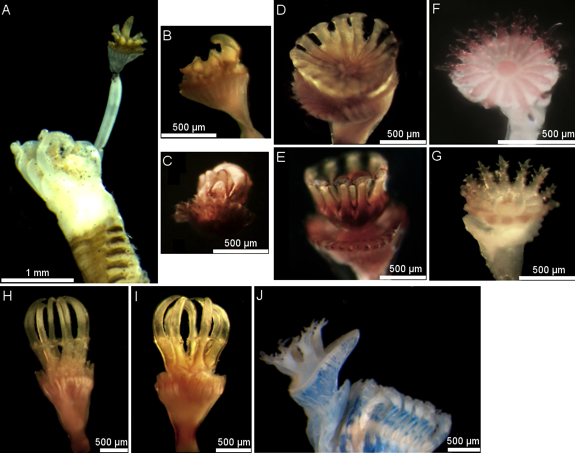


S.D. Fig. 9 (A-J). Serpulids- Close-ups of the serpulid’s opercula: (A-C) *Hydroides brachyacantha sensu lato* in Marina #2; (D-E) *Hydroides dirampha* in Marina #23; (F-G) *Hydroides elegans* in Marina #18; (H-I) *Hydroides homoceros* in Marina #33; (J) *Spirobranchus tetraceros sensu lato* in Marina #18. Photo credits: (A, J) Giorgos Chatzigeorgiou; (B-I) Aylin Ulman.


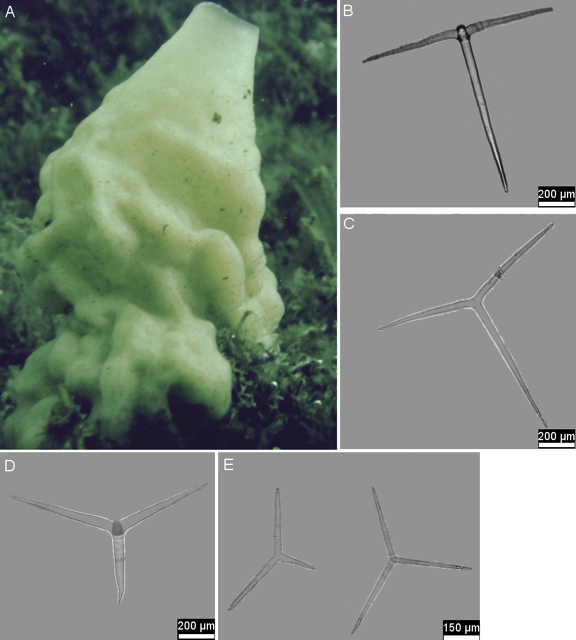


S.D. Figure 10 (A-E). Porifera: *Paraleucilla magna* (A) Live colony in Marina #24. (B) cortical tetractine; (C) subatrial triactine; (D) subatrial tetractine; (E) atrial triantine (left) and cortical triactine (right). Photo credits: (A) Aylin Ulman; (B-E) Marco Bertolino.


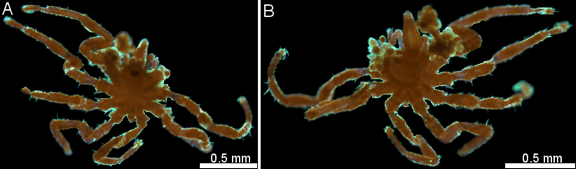


S.D. Fig. 11 (A-B). Pycnogonida: (A & B) *Achelia sawayai sensu lato* Marcus,1940, ♂ (ovigerous) from Malta in Marina #23, (A) dorsal view; (B) ventral view. Photo credits: Cengiz Koçak.
